# Metabolic analysis and identification of potential biomarkers of early-stage melanoma lung metastasis: Metabolic profiling of early-stage melanoma lung metastasis

**DOI:** 10.3724/abbs.2025136

**Published:** 2025-10-09

**Authors:** Chunying Gu, Hongyu Liu, Guangyu Jiang, Ying Lv, Jiafu Liu

**Affiliations:** 1 Departments of Laboratory Diagnosis the Fifth Affiliated Hospital of Harbin Medical University Daqing 163319 China; 2 Heilongjiang Provincial Center for Animal Disease Prevention and Control Harbin 150069 China; 3 College of Basic Medical Sciences Harbin Medical University Daqing 163319 China; 4 College of Life Sciences Northeast Agricultural University Harbin 150030 China

**Keywords:** metabolomics, metastasis, NMR, biomarker, methylxanthine, allantoin

## Abstract

Tumor cells exhibit a notable ability to adapt to constantly changing microenvironments and possess distinct metabolic traits during metastasis. This study aims to establish a melanoma lung metastasis model in mice to elucidate the metabolic mechanisms involved in early-stage metastasis prior to treatment. The male C57BL/6 mice are divided into five groups based on time intervals of 6, 24, 72, and 120 h post-injection (SKCM-M groups) of melanoma cells, as well as a normal control group (NOR group). Our results demonstrate that platelet activation mainly occurs in the initial phases of metastasis to help tumor cells survive. NMR-based metabolomics analysis of mouse lung tissues identifies distinct metabolites and pathways associated with early-stage metastasis, revealing significant alterations in energy and amino acid metabolism during tumor progression. Further analysis indicates that methylxanthine and allantoin could serve as potential biomarkers for monitoring the early progression of tumor metastasis in cancer patients, providing novel insights into early diagnostic strategies for lung metastasis.

## Introduction

Tumor metastasis is an extraordinarily complex and multifactorial phenomenon characterized by metabolic alterations across multiple organs and is intricately intertwined with diverse biological processes. Approximately 90% of cancer-related fatalities are caused by metastasis [1], with approximately 20% of cases presenting with distant metastasis at the time of clinical diagnosis [2]. Metastasis progression is intricate and comprises multiple steps, which are dependent not only on the intrinsic characteristics of tumor cells but also on their interaction with the surrounding microenvironment [ 3, 4] . This interaction profoundly influences cancer progression and metastasis.

The viability of tumor cells in the bloodstream during metastasis is crucial, with tumor-educated platelets (TEPs) identified as functional cells by exhibiting a distinctive tumor-promoting phenotype. When platelets are triggered by tumor cells, they become activated and form a protective barrier around the tumor cells, helping tumor cells evade the host’s immune response [5]. Scientists postulate that tumor cell-induced platelet activation is a pivotal factor in the process of hematogenous metastasis in cancer [ 6, 7] . One mechanism involves the interaction between the podoplanin protein (PDPN) on the surfaces of various tumor cells and the C-type lectin-like receptor 2 (CLEC-2) on platelets, which activates platelets and promotes tumor metastasis [ 8– 10] .

To survive and thrive in new organs, the metabolic characteristics of metastatic cells, including transcriptional regulation, translation, and enzyme utilization, are continually adjusted [11]. Additionally, many secondary tumors develop metabolic profiles distinct from those of primary tumors, enhancing their adaptation to the new environment [12]. Metabolism serves as the pivotal hub of cellular existence, intricately connecting various elements such as the microenvironment, phenotype, signal transduction, and genetic background [ 13, 14] . Therefore, metabolic profiling analysis can provide deeper insights into the mechanisms and pathways underlying disease progression [15]. Metabolomics, a valuable tool for examining biological metabolic changes, offers theoretical and technical support for the early detection and treatment of diseases.

Although significant progress has been made in identifying biomarkers for tumor metastasis, research still faces persistent limitations and challenges. Relying solely on clinical imaging cannot detect pathological changes that precede the formation of solid tumors [ 16, 17] . Metabolic changes are often subtle and dynamic during the early stage of lung metastasis. Most studies utilize single time-point samples, potentially missing critical metabolic events. While the metabolism of lung metastases and their surrounding microenvironment plays a key role in metastatic progression and colonization, current research primarily focuses on tumor cell metabolism, often overlooking the contributions of stromal cells such as fibroblasts and platelets. Consequently, the detection efficiency of existing biomarkers for early metastasis remains limited. Therefore, there is an urgent need to develop highly sensitive, easy-to-use, and reliable biomarkers for accurately evaluating the progression of tumor cell metastasis.

In the present study, we established a mouse cutaneous melanoma (SKCM-M) model to mimic early lung metastasis. Initially, H&E staining was performed on lung tissue pathology slices to observe structural alterations around the pulmonary vasculature. NMR metabolomics analysis was then conducted to examine the metabolic profiles of the five groups, namely, the 6-h, 24-h, 72-h, and 120-h post-inoculation groups, and compared with the control group (NOR). Furthermore, multivariate receiver operating characteristic (ROC) curve analysis was used to identify potential biomarkers in mouse lung tissues that accurately distinguish successful colonization during early-stage metastasis. Our research provides new insights into the metabolic mechanisms involved in early-stage metastasis and presents a promising tool for clinical diagnosis and treatment.

## Materials and Methods

### Cell culture

The B16-F10 mouse melanoma cell line was maintained in our laboratory. The cells were cultured in DMEM (HyClone, Logan, USA) supplemented with 10% fetal bovine serum (FBS), 100 U/mL penicillin, and 100 μg/mL streptomycin at a constant temperature of 37°C with 5% CO _2_ in an incubator. The cells were harvested via digestion with 0.25% trypsin-EDTA (HyClone, Logan, USA) when the confluency reached 85%–95%.

### Animal handling

Male C57BL/6 mice (20 ± 2 g) were obtained from Changchun Yisi Experimental Animal Technology Co., Ltd. (Changchun, China) with Certificate No. SCXK [Ji]2018–0007 and were certified as specific pathogen-free. All experimental protocols strictly adhered to the regulations for laboratory animal care and use established by the Experimental Animal Ethics Committee of Harbin Medical University.

First, we needed to identify optimal modelling conditions to ensure stable lung metastasis while adhering to animal ethics guidelines. Three groups of mice were injected via the tail vein with varying doses of B16F10 cells (3 × 10 ^6^/150 μL, 1 × 10 ^6^/150 μL, and 5 × 10 ^5^/150 μL) to establish a mouse model of cutaneous melanoma (SKCM-M) lung metastasis. After being bred for 7, 14, and 21 days, the mice gradually exhibited relevant symptoms such as weight loss, muscle atrophy, decreased fat, emaciation, and reduced activity. The mice were then dissected to observe the growth and metastasis of melanoma to determine the most suitable modelling dose and colonization location [ 18, 19] . Finally, the optimal inoculation dose of 1 × 10 ^6^/150 μL B16F10 cells was selected for this study.

An early-stage melanoma lung metastasis mouse model was constructed to investigate the influence of platelets and metabolic alterations in lung tissue during early malignant tumor metastasis. Fifty mice were divided into five groups, with 10 mice in each group. The control group (NOR) received a tail vein injection of an equal volume of 0.9% sodium chloride solution, whereas the remaining four groups (6-h, 24-h, 72-h, and 120-h groups) were injected with B16F10 cells (1 × 10 ^6^/150 μL) via the tail vein. The mice were dissected at 6-h, 24-h, 72-h, and 120-h post-injection, and both lung tissue and blood samples were collected to analyze melanoma growth and early metastasis.

### H&E staining assay

Paraffin-embedded mouse lung tissue samples were obtained and sectioned. The sections were then deparaffinized and stained with hematoxylin and eosin (H&E) to visualize pathological changes surrounding the blood vessels in the lung tissue. The results of H&E staining are shown as color images at 40× and 200× magnification.

### Platelet analysis

Platelet isolation was conducted at room temperature (15–37°C), protected from light, and refrigeration or freezing was avoided. Platelet-rich plasma was obtained by centrifuging the sample at 200–300 *g* for 15 min at room temperature, following an anticoagulant-to-blood ratio of 1:9. The plasma was then centrifuged at 800 *g* for 10 min to collect the platelet pellets. Platelet function tests were performed directly using a platelet aggregation function detection kit (Helena Laboratories, Beaumont, USA) with turbidimetric methods. *In vitro*, platelet aggregation was simulated, with increases in optical density reflecting the intensity of platelet aggregation. Variations in absorbance were used to quantify and document the degree of platelet aggregation.

### Quantitative RT-PCR

Total RNA was extracted from lung tissue and platelets using RNAiso Plus (Takara, Shiga, Japan). The RNA concentration was measured with a NanoDrop One spectrophotometer (Thermo Fisher Scientific, Waltham, USA). The RNA was subsequently reverse transcribed into cDNA using a reverse transcription kit (HaiGene, Harbin, China). The cDNA was then quantified using SYBR® Green Real-time PCR Master Mix (QPK-201; TOYOBO, Tokyo, Japan) on a Rotor-Gene Q real-time PCR analyzer (QIAGEN, Hilden, Germany), with *18S* serving as an internal reference. Details of the primers used in the experiments are shown in Supplementary Table S1.

### Western blot analysis

Protein factors from tissues and platelets were analyzed using western blot analysis. Breifly, the extracts were separated by 10%–15% SDS-PAGE and transferred onto PVDF membranes. After being blocked with 5% non-fat milk at room temperature for 60 min, the membranes were incubated overnight at 4°C with specific primary antibodies, including anti-CLEC-2 (Abcam, Cambridge, UK), anti-AKT (Proteintech, Wuhan, China), anti-phospho-AKT (Proteintech), CD31 polyclonal antibody (Proteintech), VEGFA polyclonal antibody (Proteintech), INPP5D polyclonal antibody (Proteintech), phospho-PI3 kinase p85 (CST, Danvers, USA), and PI3 kinase p85 (Novus, Littleton, USA) antibodies. The membrane was then incubated with horseradish peroxidase-conjugated secondary antibody solution (Beyotime Biotechnology, Shanghai, China) for 1 h at room temperature. Specific protein signals were visualized using the UVP Gel Studio PLUS gel imaging system (Analytik Jena AG, Jena, Germany), followed by quantitative analysis with ImageJ software.

### ELISA

The concentrations of methylxanthine and allantoin in the lung tissue were measured using ELISA kits (COIBO BIO, Shanghai, China) according to the manufacturer’s instructions. Lung tissues were harvested, immediately frozen in liquid nitrogen, and stored at –80°C until analysis. For assay preparation, the tissues were weighed and homogenized in cold phosphate-buffered saline (PBS; pH 7.4) containing protease inhibitors using a mechanical homogenizer. The homogenates were then centrifuged at 12,000 *g* for 15 min at 4°C to remove cellular debris, and the supernatants were collected for ELISA. Biotinylated detection antibodies specific for methylxanthine or allantoin, followed by incubation with streptavidin-conjugated horseradish peroxidase (HRP), were used for the reaction. Colorimetric detection was performed using a TMB substrate, and the absorbance was measured at 450 nm with a microplate reader (Molecular Devices, Shanghai, China). Metabolite concentrations were quantified on the basis of standard curves generated with known concentrations ( Supplementary Figure S1). All the measurements were performed four times.

### NMR sample preparation

Lung tissue samples were collected and promptly encapsulated after thorough washing with prechilled PBS and then stored at –80°C. Prechilled 100% methanol was mixed with 100 mg of tissue from each sample at a ratio of 4 mL per gram of tissue and homogenized for 5 min. Prechilled deionized water was then added at a ratio of 2.85 mL per gram of tissue, followed by homogenization for 2 min. Next, prechilled chloroform was added at a ratio of 4 mL per gram of tissue, and the mixture was homogenized for another 2 min. The solution was vortexed for 5 min and centrifuged at 16,000 *g* at 4°C for 10 min to separate the organic and aqueous phases [ 20– 22] . The aqueous phase was then subjected to methanol evaporation using a nitrogen evaporator, followed by freezing in a lyophilizer to obtain a powder. The powdered samples were then dissolved in NMR buffer [10% D _2_O, 50 mM PBS, and 1 mM 3-(trimethylsilyl) propionic-2,2,3,3-d4 acid (TSP)] and transferred into 5 mm NMR tubes for NMR experiments [ 23, 24] .

### NMR measurements and data preprocessing

NMR experiments were conducted at 298K on a Bruker Avance III HD 850 MHz spectrometer equipped with a TCI cryoprobe (Bruker BioSpin, Ettlingen, Germany). One-dimensional (1D) ^1^H-NMR spectra were acquired using the pulse sequence NOESYGPPR1D with a relaxation delay (RD) of 4 s, short delay (t) of 4 μs, mixing time (τm) of 10 ms, and spectral width of 20 ppm.

The Chenomx NMR Suite 10.0 software package (Chenomx, Edmonton, Canada) was used for phase adjustment, baseline correction, and calibration of each 1D ^1^H-NMR spectrum [25]. The TSP methyl groups were set at 0 ppm for chemical shift calibration. The spectral region of 0.5–9.5 ppm was divided into 0.01 ppm units for further multivariate statistical analysis. Signal assignment of the NMR spectra was performed according to the current literature [ 26, 27] and the Analyzer module in the Chenomx NMR Suite. The region of 4.7–4.9 ppm corresponding to the water peak was excluded to eliminate its interference with the baseline. Data normalization was applied to minimize the influence of sample concentration variations on the analysis.

### Metabolomic analysis

The normalized data were imported into SIMCA 14.1 software (Umetrics, Umea, Sweden) for multivariate statistical analysis, including unsupervised principal component analysis (PCA) and supervised orthogonal partial least squares-discriminant analysis (OPLS-DA), which were employed to examine metabolic profiles and determine metabolic differences between groups. PCA was used to identify the primary sources of variation and to cluster samples according to their similarities, thereby simplifying data complexity. OPLS-DA was applied to maximize differentiation between groups while minimizing variability within each group. Significant metabolites were identified by the OPLS-DA model using variable importance in projection (VIP) scores with a threshold of VIP > 1.

Significantly altered metabolic pathways between groups were identified via the MetaboAnalyst 6.0 web server ( http://www.MetaboAnalyst.ca, accessed on 10 March 2024). The significance of metabolite enrichment within specific pathways was assessed via pathway enrichment analysis on the basis of the concentrations of all identified metabolites. *P*  < 0.05 was used to represent significant enrichment. Pathway topological analysis was conducted to assess the importance of the identified pathways, with the pathway impact value (PIV) used to evaluate the impact of each metabolite on specific metabolic pathways. Higher PIV values indicate greater pathway importance. In this study, metabolic pathways with a *P* value < 0.05 and PIV > 0.1 were considered significant, contributing to the comprehension of potential metabolic mechanisms associated with inter-group metabolic disparities.

Multivariate receiver operating characteristic (ROC) curve analysis was performed using MetaboAnalyst 6.0 on mouse lung tissue samples to evaluate the relevance of metabolites in significantly altered metabolic pathways. The area under the ROC curve (AUC) was calculated using a logistic regression algorithm to assess the predictive performance of the selected biomarkers. This analysis aimed to identify potential biomarkers at the 24-h and 120-h stages. The results from univariate and multivariate analyses were combined with the ROC analysis of metabolites to select potential biomarkers. The criteria for choosing these potential biomarkers included Log _2_ (fold change) of metabolite concentration > 0.6 or < –1, VIP > 1, and AUC > 0.7. This approach allowed us to identify the most relevant metabolites for differentiating between various stages of tumor cell implantation before and after successful seeding. This step is crucial for understanding tumor biology and studying the processes of tumor growth and dissemination [28].

### Statistical analysis

The experimental data are presented as the mean ± SD. Statistical analysis was performed via GraphPad Prism 8.0.2 (GraphPad Software, La Jolla, USA). Pairwise comparisons among the five groups of mouse lung tissues were conducted using one-way analysis of variance (ANOVA) followed by Tukey’s multiple comparison test. *P* value less than 0.05 was considered as statistically significant. Metabolites with VIP > 1 and *P*  < 0.05 were considered characteristic metabolites.

## Results

### Construction of the melanoma lung metastasis model

The initial goal of this study was to establish a stable mouse model of melanoma lung metastasis to understand the biological mechanisms underlying tumor metastasis and to investigate the metabolic characteristics during the early stages of lung metastasis. The mouse melanoma cell line B16F10 used in this study is derived from spontaneous tumor cells of C57BL/6 mice and is particularly suitable for constructing tumor metastasis models because of its rapid proliferation, strong invasiveness, and high tumor formation rate.

**Figure 1:**
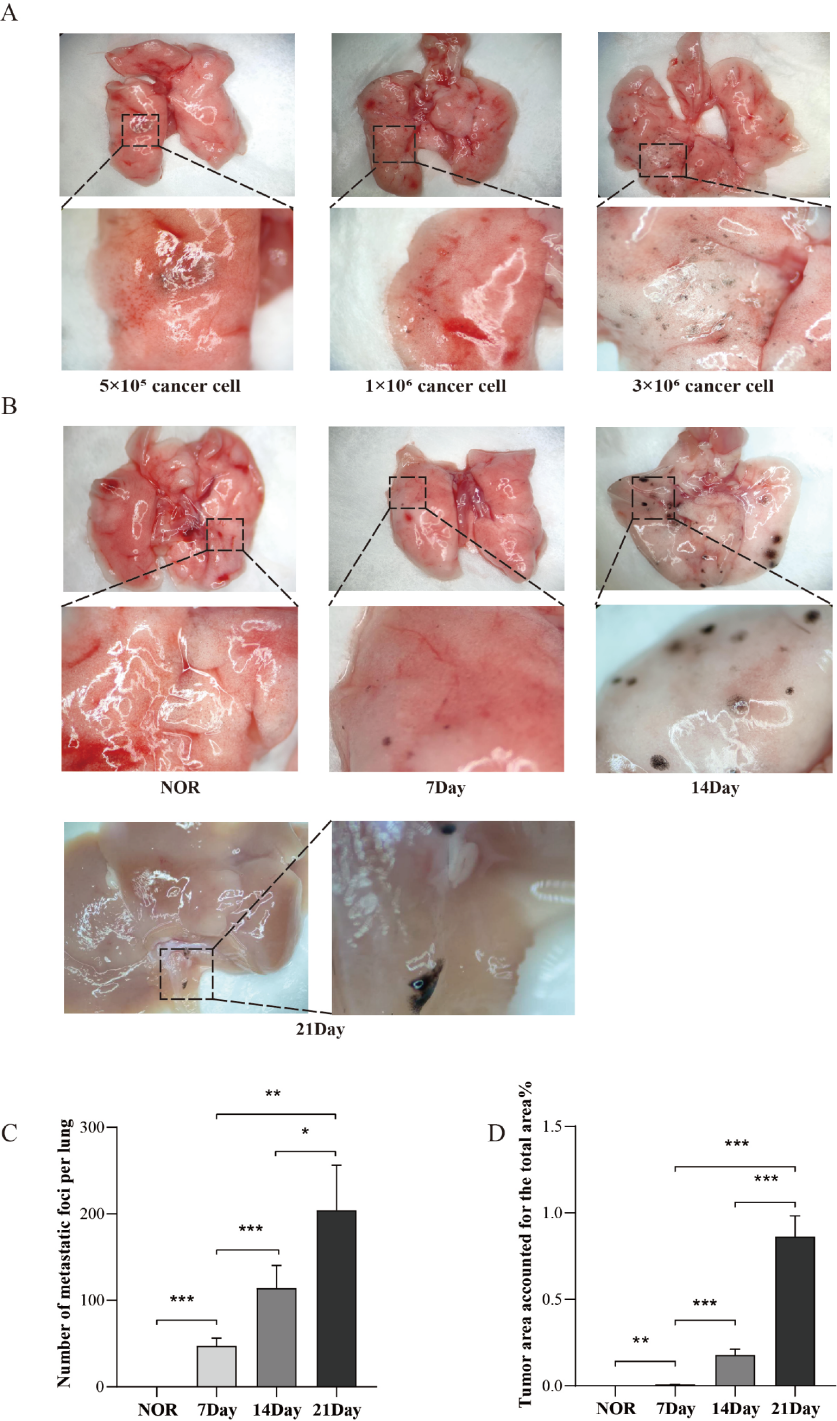
Figure 1 Establishment of the melanoma lung metastasis model (A) Identification of tumor cell concentrations: 5 × 105 B16F10 cells, 1 × 106 B16F10 cells, and 3 × 106 B16F10 cells. The samples were dissected on the 7th day. (B) Metastatic foci on the lung surface (NOR, 7, and 14 days) and inside the liver (21 days). (C) The number of lung tissue metastatic foci at NOR, 7 days, 14 days, and 21 days. n = 8. (D) The relative area of metastatic foci at NOR, 7 days, 14 days, and 21 days. n = 7. Original magnification: 1× or 4×, respectively. *P < 0.05, **P < 0.01, and ***P < 0.001.

To identify the optimal inoculation dose for the experiment, three different concentrations of B16F10 cells were administered to the mice. Seven days later, the mice were dissected, and the results revealed that when inoculated with 5 × 10 ^5^ B16F10 cells, there were fewer lung metastases and a lower tumor formation rate. At a dose of 1 × 10 ^6^ B16F10 cells, a moderate number of lung metastases were observed, with a tumor formation rate reaching 90%, making it easier to count the metastatic foci. However, when 3 × 10 ^6^ B16F10 cells were administered, the number of metastatic foci in the lungs became too large to count accurately. Additionally, the mice exhibited severe symptoms, including respiratory distress, decreased appetite, weight loss, and a high mortality rate. These results did not meet the requirements of the experiment ( Figure 1A). As a result, 1 × 10 ^6^ B16F10 cells were chosen as the optimal inoculation dose for subsequent experiments.

To determine the colonization site and early-stage time points of tumor metastasis, 1 × 10 ^6^ B16F10 cells were injected through the tail vein, and the mice were dissected at different time points. By day 7, a few small melanoma metastases were detected in the lungs, with no evidence of metastasis in other tissues or organs. By day 14, both the number and size of metastatic lesions in the lungs were increased. By day 21, there was a significant expansion in the area of lung metastases, with the mice nearing death. Additionally, melanoma metastases were observed on the liver surface ( Figure 1B). The metastatic foci on the lung surface at 21 days were too large and are not shown in Figure 1B. We also quantified the number of tumor metastases in the lung tissue across the three groups ( Figure 1C) and calculated the percentage of metastatic involvement in the entire lung area ( Figure 1D). These findings suggested that melanoma cells successfully colonized lung tissue and began proliferating within 7 days of inoculation, with metastatic foci in other organs appearing only until the late stages of metastasis.

### Early-stage melanoma cell aggregation and angiogenesis around pulmonary blood vessels during metastasis

**Figure 2:**
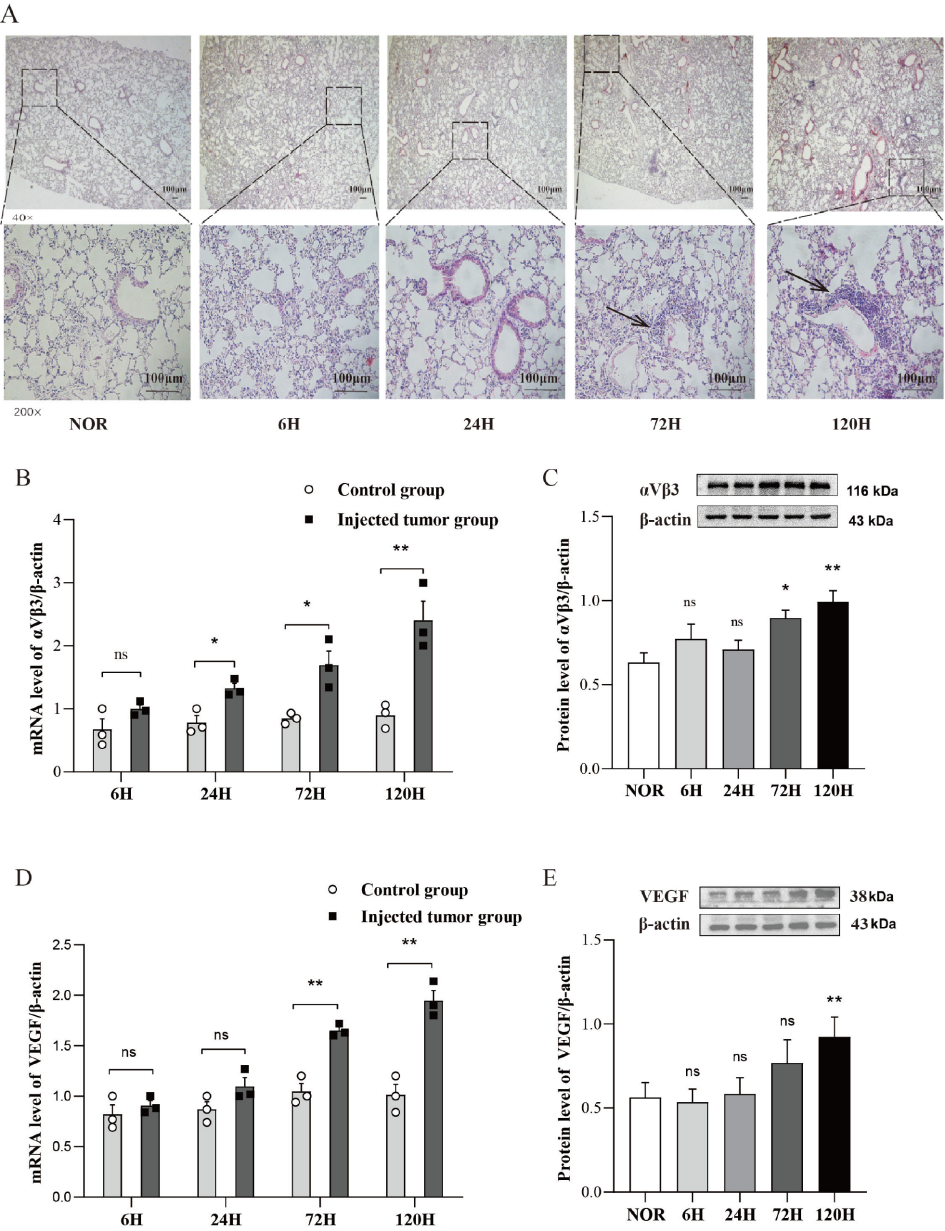
Figure 2 Melanoma cells aggregate around pulmonary blood vessels and promote angiogenesis in the early stage (A) H&E staining of mouse lungs was performed to detect tumor metastasis. The arrows indicate the aggregation of tumor cells around blood vessels. Original magnification: 40× or 200×, respectively. (B) qPCR analysis of αVβ3 mRNA level in lung tissue. n = 4. (C) Western blot analysis of αVβ3 expression in lung tissue. n = 4. (D) qPCR analysis of VEGF mRNA level in lung tissue. n = 4. (E) Western blot analysis of VEGF expression in lung tissue. n = 4. ns, not significant. *P < 0.05, **P < 0.01. Representative blots are shown here, and all the data from multiple biological replicates are displayed in Supplementary Figure S1.

H&E staining of lung tissue sections was used to observe structural changes around pulmonary blood vessels. Compared with that in the NOR control group, the aggregation of tumor cells around blood vessels significantly increased in the 72-h and 120-h groups, whereas minimal differences were observed in the 6-h and 24-h groups ( Figure 2A). Although no abnormalities were visible on the lung surface, H&E-stained sections clearly revealed successful melanoma cell colonization at 72 h, with further progression by 120 h. Additionally, the mRNA and protein expression levels of VEGF and αVβ3 in mouse lung tissue were significantly elevated in the 72-h and 120-h groups ( Figure 2B–E). These findings indicated that melanoma cells were successfully colonized by 72 h, followed by aggregation, growth, and the initiation of angiogenesis around blood vessels. Preliminary experiments revealed that a few metastatic foci were observed on the lung surface on the 7th day after inoculation ( Figure 1B). However, H&E staining revealed that the melanoma cells clearly aggregated around the pulmonary blood vessels by the 5th day. Consequently, lung tissues were collected at 6, 24, 72, and 120 h post-inoculation in the subsequent assays to mimic early-stage metastatic events.

### Platelet activation in the early stage of melanoma lung metastasis

**Figure 3:**
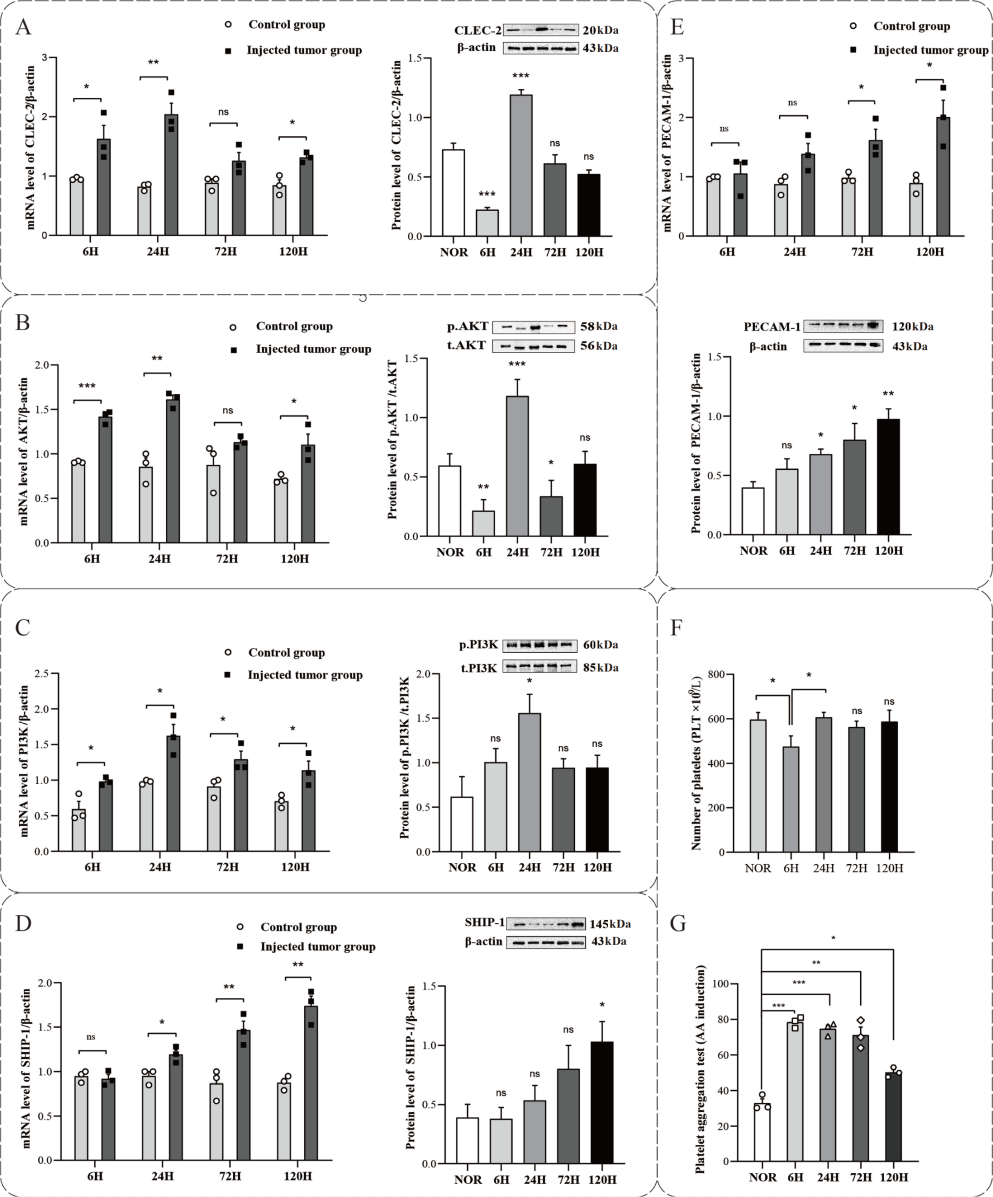
Figure 3 Tumor cell-induced platelet activation in early-stage melanoma lung metastasis (A–E) The mRNA and protein expression levels of CLEC-2, AKT, PI3K, SHIP and PECAM-1 in platelets were detected by qPCR and western blot analysis. n = 4. (F) Platelet count (PLT). n = 5. (G) Platelet function tests revealed continuous activation at 6 h, 24 h, and 72 h. n = 5. ns, not significant. *P < 0.05, **P < 0.01, ***P < 0.001.

This study used the SKCM-M mouse melanoma lung metastasis model to investigate platelet activation during the early stages of melanoma cell metastasis. The mRNA and protein expression levels of platelet-associated factors were analyzed in the NOR group, as well as at 6, 24, 72, and 120 h after inoculation with B16F10 cells. qPCR and western blot analysis results revealed an increase in the expressions of the platelet-activating proteins CLEC-2, PI3K, and AKT at 24 h ( Figure 3A–C), whereas the expressions of SHIP-1 and PECAM-1, which inhibit platelet activation, increased after 72 h ( Figure 3D,E), indicating the suppression of platelet activation. These findings suggested that platelet activation reached its peak at 24 h after B16F10 cell inoculation and that platelet inhibitory proteins were upregulated following successful tumor colonization, thereby promoting platelet recovery to a resting state. These data imply that platelet activation predominantly occurs during the early stages of tumor cell metastasis, facilitating tumor cell survival, dissemination, and colonization.

Additionally, platelet indicators in the blood were assessed during the early stages of melanoma lung metastasis. Compared with those in the NOR group, both the platelet count (PLT) and the platelet pressure decreased at 6 h postinoculation across the 6-h, 24-h, 72-h, and 120-h groups ( Figure 3F). These findings indicated that tumor cells consume a significant quantity of platelets during the early stages of their entry into the blood circulation. Notably, the platelet width (PDW) and mean platelet volume (MPV) remained unchanged, indicating that the decrease in platelet count was not due to bone marrow lesions, inflammation, or sepsis but rather to the activation of platelets, which form a protective barrier around tumor cells to evade immune attacks and are consumed as tumor cells attach to the vessel wall. Arachidonic acid functional tests of platelets further demonstrated sustained activation within the first 6 to 24 h, with a gradual decline from 72 h and a return to baseline by 120 h ( Figure 3G). These results further confirmed the role of platelet activation in the early stages of tumor cell metastasis and its importance in supporting tumor cell survival.

### NMR spectra of aqueous extracts of mouse lung tissue

**Figure 4:**
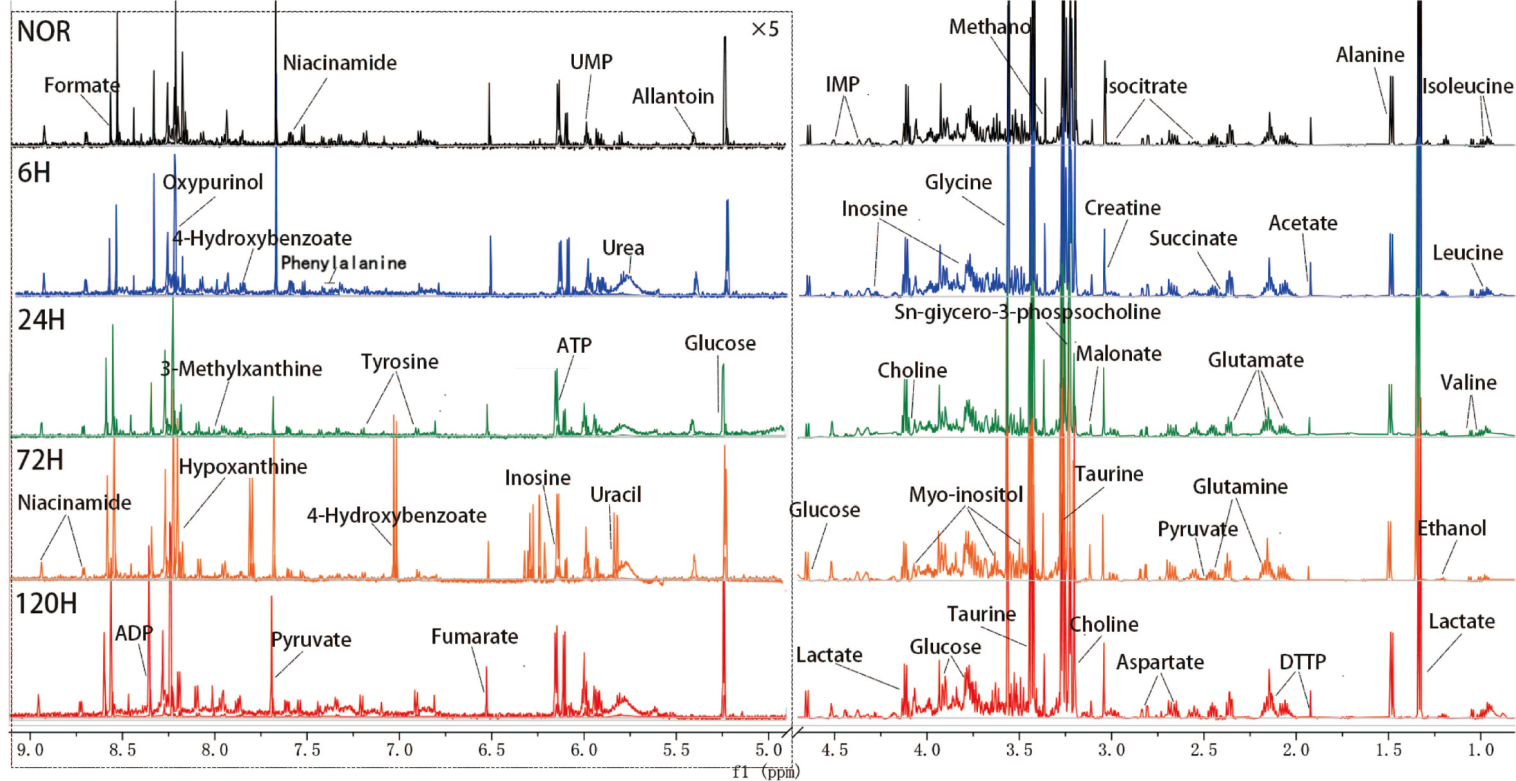
Figure 4 600 MHz 1D ^1^H-NMR spectra recorded on aqueous extracts from five groups of mouse lung tissue The spectra span the regions of 0.5–4.7 ppm and 4.9–9.5 ppm, with the water peak between 4.7–4.9 ppm removed. The region from 4.9–9.5 ppm has been magnified five times for clarity.

Typical 1D ^1^H-NMR spectra of aqueous extracts from the NOR, 6-h, 24-h, 72-h, and 120-h groups of mouse lung tissue are shown in Figure 4. Chenomx NMR Suite 10.0 software and the KEGG database were used to assign the signals and confirm the identified metabolites. A total of 40 metabolites were assigned and are summarized in Supplementary Table S2. Direct observation revealed that the chemical shifts of the peaks in these five spectra were largely consistent, and there were notable differences in peak intensity. The data were then analyzed using multivariate analysis methods such as PCA and OPLS-DA.

### Multivariate statistical analysis of the metabolic profiles

**Figure 5:**
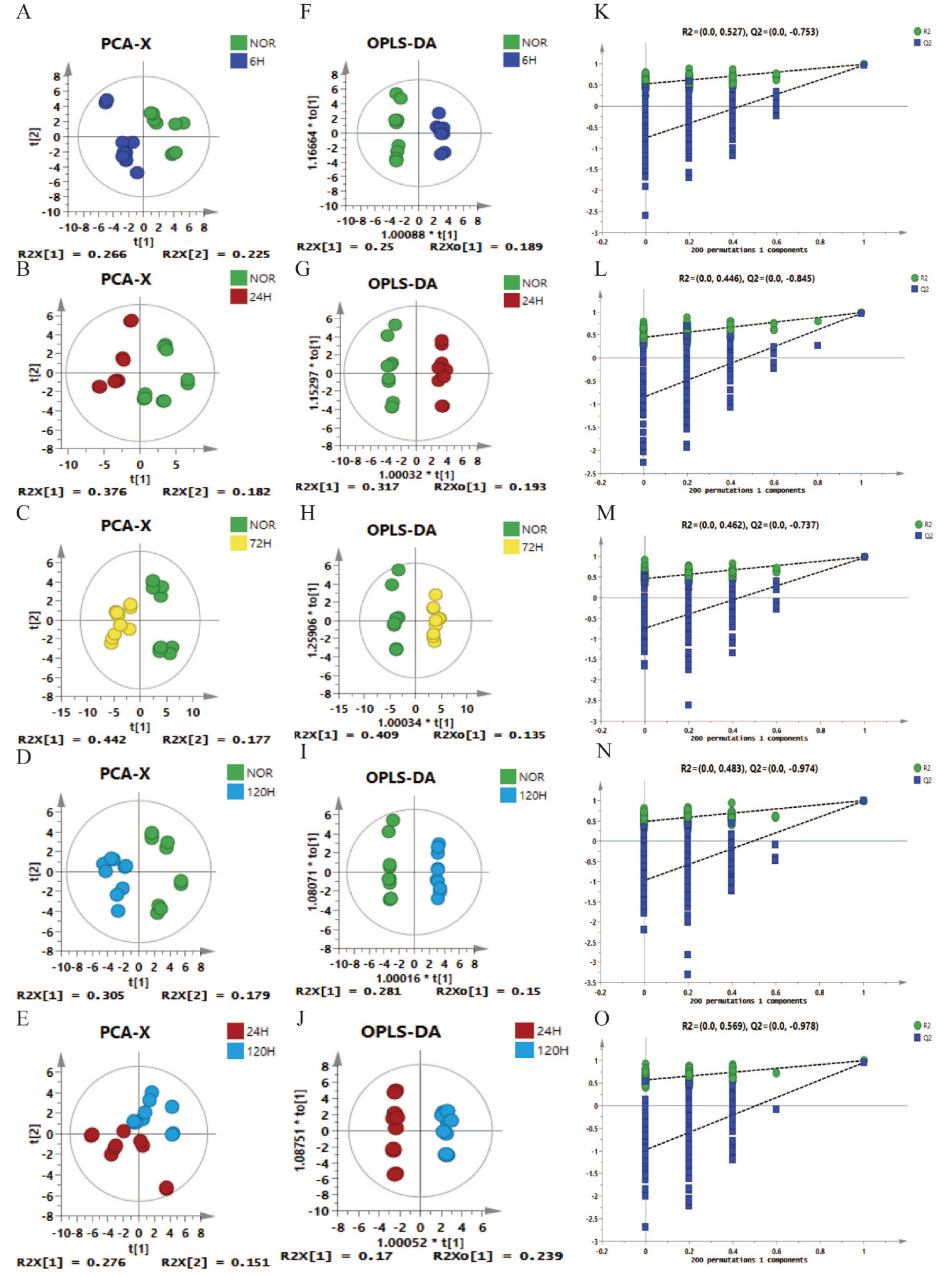
Figure 5 Multivariate statistical analysis of 1D ^1^H-NMR spectra from five sets of mouse lung tissue (A–E) PCA score plots showing the metabolic differences between the NOR group and the 6-h, 24-h, 72-h, and 120-h groups and between the 24-h and 120-h groups. (F–J) Score plots of OPLS-DA models for the five groups, with ellipses indicating 95% confidence intervals. (K–O) Cross-validation plots among the five groups, assessing the reliability of the OPLS-DA models through 200 iterations of random permutation tests.

Multivariate statistical analyses were performed on the NMR spectral data using SIMCA software (Ver.14.2). PCA was employed to reduce the complexity of the datasets to a few components to identify the distinct metabolic features and outliers that could impact the validity of the analysis. In particular, PCA was used to gain insight into the metabolic profiles of the five groups of mouse lung tissues. Each dot in the PCA score plot represents the metabolic pattern of each lung tissue sample, with closer clustering of dots representing more similar metabolic patterns. The PCA score plots captured the metabolic differences between the NOR group and the 6-h, 24-h, 72-h, and 120-h groups, as well as between the 24-h and 120-h groups in early tumor metastatic lung tissue ( Figure 5A–E). A greater dispersion was observed in the NOR group than in the 72-h and 120-h groups ( Figure 5C,D). Several potential factors, such as the biological variability in the control animals, tumor-induced metabolic convergence, sample size and group heterogeneity, and experimental variation, may have contributed to the greater dispersion in the PCA plots.

To further emphasize the metabolomic differences between the NOR and SKCM-M groups, supervised OPLS-DA (Orthogonal Partial Least Squares Discriminant Analysis) was performed on the NMR datasets. This analysis enhanced the metabolic distinctions between the groups, as illustrated in the OPLS-DA score plots for the NOR vs 6-h, NOR vs 24-h, NOR vs 72-h, NOR vs 120-h, and 24-h vs 120-h comparisons ( Figure 5F–J). The reliability of the OPLS-DA model was confirmed through a 200-cycle response permutation test (RPT) ( Figure 5K–O). The Q ^2^ regression line intercepts were negative, and the remaining R ^2^ values were located below the right original points, indicating that the original OPLS-DA models were effective. The R ^2^Y and Q ^2^Y values for the five sets of OPLS-DA models, which represent the model interpretation rate and predictive ability, respectively, were as follows: R ^2^Y = 0.993 and Q ^2^Y = 0.969 for the NOR group vs the 6-h group; R ^2^Y = 0.993 and Q ^2^Y = 0.975 for the NOR group vs the 24-h group; R ^2^Y = 0.99 and Q ^2^Y = 0.974 for the NOR group vs the 72-h group; R ^2^Y = 0.998 and Q ^2^Y = 0.987 for the NOR group vs the 120-h group; and R ^2^Y = 0.995 and Q ^2^Y = 0.954 for the 24-h group vs the 120-h group, indicating that these established OPLS-DA models were robust and reliable.

### Identification of differential and significant metabolites

**Figure 6:**
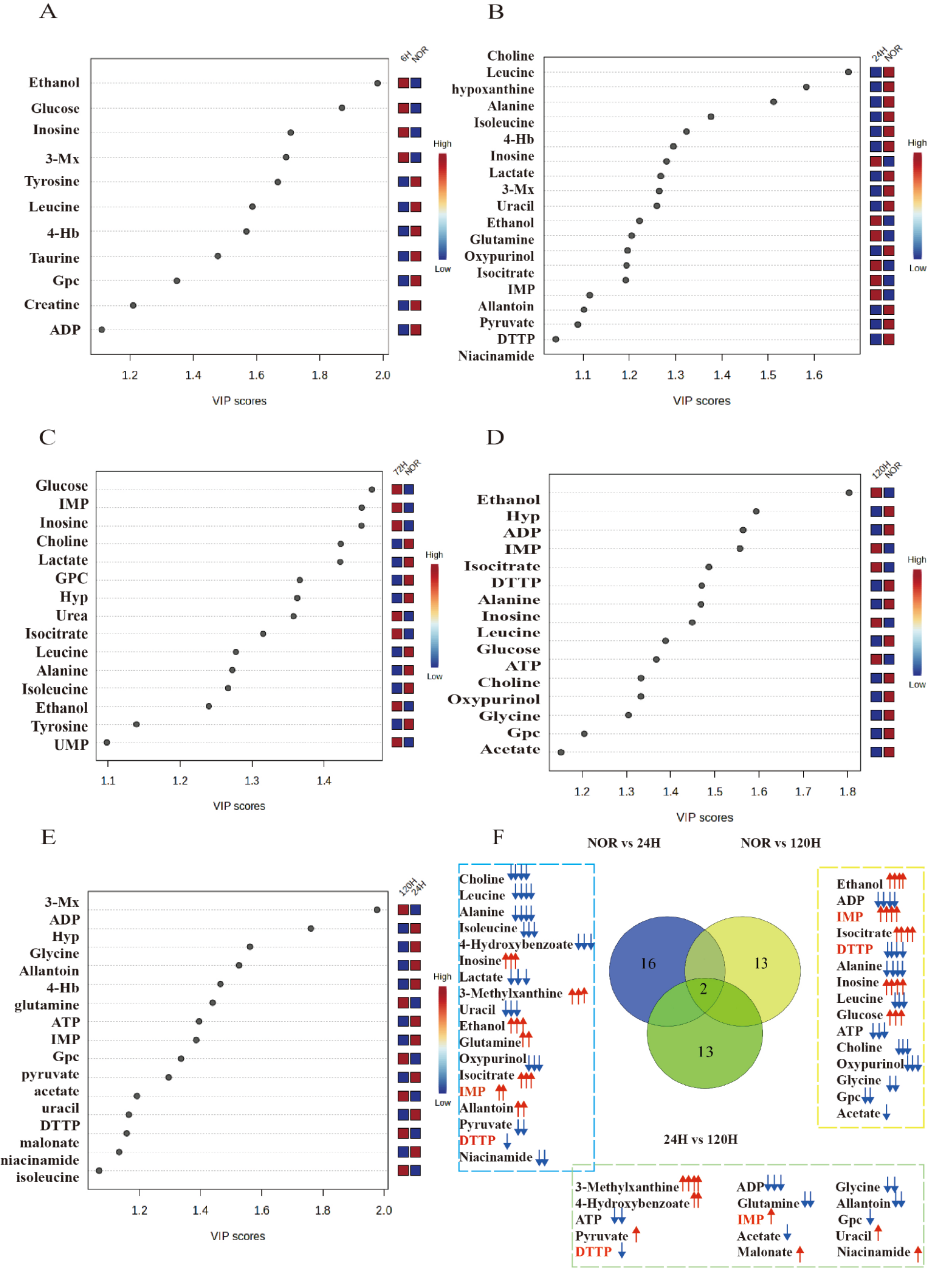
Figure 6 Identification of differential and important metabolites VIP score-ranking plots of important metabolites identified from pairwise comparisons of OPLS-DA models for (A) NOR vs 6-h, (B) NOR vs 24-h, (C) NOR vs 72-h, (D) NOR vs 120-h, and (E) 24-h vs 120-h groups. (F) Venn diagram of characteristic metabolites. The significant metabolites were identified from the OPLS-DA models (VIP > 1) and univariate analyses (P < 0.05). Upward arrows indicate a positive difference (a > b), while downward arrows indicate a negative difference (a < b).

The reliability of the OPLS-DA models was validated by comparisons between NOR and 6-h, NOR and 24-h, NOR and 72-h, NOR and 120-h, and 24-h and 120-h groups using cross-validation plots obtained from response permutation tests (RPTs). With the criterion of VIP > 1, 11 significant metabolites were identified from the NOR vs 6-h comparison, 19 significant metabolites were identified from the NOR vs 24-h comparison, 15 significant metabolites were identified from the NOR vs 72-h comparison, 16 significant metabolites were identified from the NOR vs 120-h comparison ( Figure 6A–E), and 17 significant metabolites were identified from the 24-h vs 120-h comparison.

One-way ANOVA and Tukey’s multiple comparison test were subsequently conducted to statistically compare the relative levels of metabolites among the NOR, 6-h, 24-h, 72-h, and 120-h groups. Using the criterion of *P*  < 0.05, we identified 16 differentially abundant metabolites from the NOR vs 6-h comparison, 23 differentially abundant metabolites from the NOR vs 24-h comparison, 28 differentially abundant metabolites from the NOR vs 72-h comparison, 19 differentially abundant metabolites from the NOR vs 120-h comparison, and 15 differentially abundant metabolites from the 24-h vs 120-h comparison ( Supplementary Table S3). This analysis enabled us to identify important metabolites that showed significant changes among the different groups, thereby further understanding metabolic changes related to early tumor metastasis. Heatmaps were constructed to illustrate the relative levels of the identified metabolites in the lung tissues from the five groups (NOR, 6-h, 24-h, 72-h, and 120-h) to visualize the changes in metabolite levels ( Supplementary Figure S3).

Furthermore, we applied two criteria, VIP > 1 and *P*  < 0.05, to identify characteristic metabolites in lung tissues. Specifically, we identified 11, 18, 15, 15, and 15 characteristic metabolites for pairwise comparisons of NOR vs 6-h, NOR vs 24-h, NOR vs 72-h, NOR vs 120-h, and 24-h vs 120-h, respectively. Notably, two common characteristic metabolite changes were observed by comparing and identifying the characteristic metabolites of the NOR vs 24-h, NOR vs 120-h, and 24-h vs 120-h groups, namely, an increase in inosine monophosphate (IMP) and a decrease in deoxythymidine triphosphate (DTTP) ( Figure 6F).

### Significant metabolic pathways during early-stage tumor metastasis

**Figure 7:**
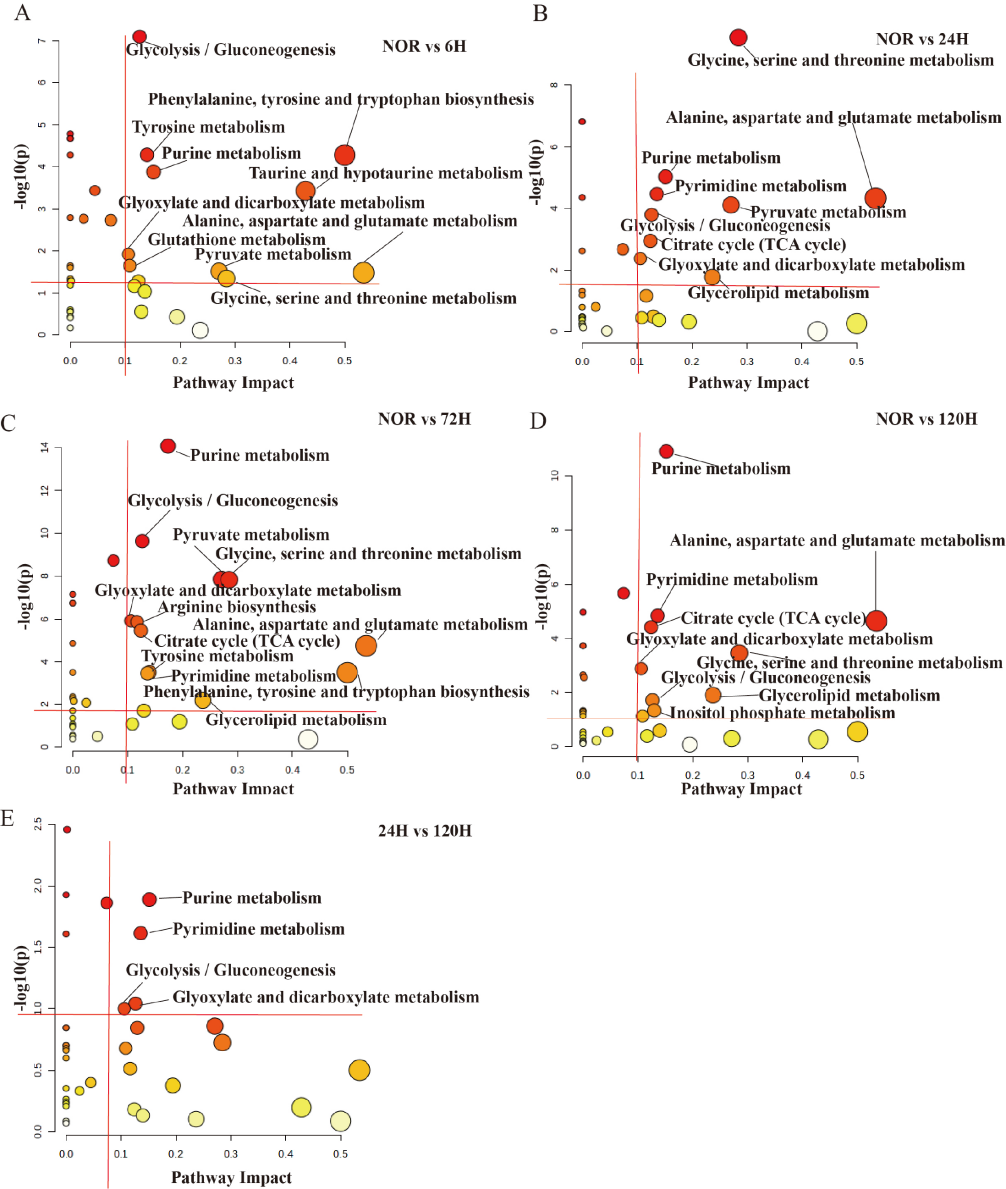
Figure 7 Identification of metabolic pathways from pairwise comparisons of the five sets (A) NOR vs 6-h. (B) NOR vs 24-h. (C) NOR vs 72-h. (D) NOR vs 120-h. (E) 24-h vs 120-h. Using the pathway analysis module provided by the MetaboAnalyst 6.0 web server, significant changes in metabolic pathways were identified with PIV > 0.1 and P < 0.05.

We then conducted an extensive analysis to identify significantly altered metabolic pathways (referred to as significant pathways) using the KEGG database and the MetaboAnalyst 6.0 web server with two criteria: a pathway impact value (PIV) > 0.1 and *P*  < 0.05 ( Figure 7 and Supplementary Table S4). In the pairwise comparisons of NOR vs 6-h, NOR vs 24-h, NOR vs 72-h, NOR vs 120-h, and 24-h vs 120-h, we identified 10, 9, 12, 9, and 4 significant pathways, respectively ( Figure 7A–E).

**Figure 8:**
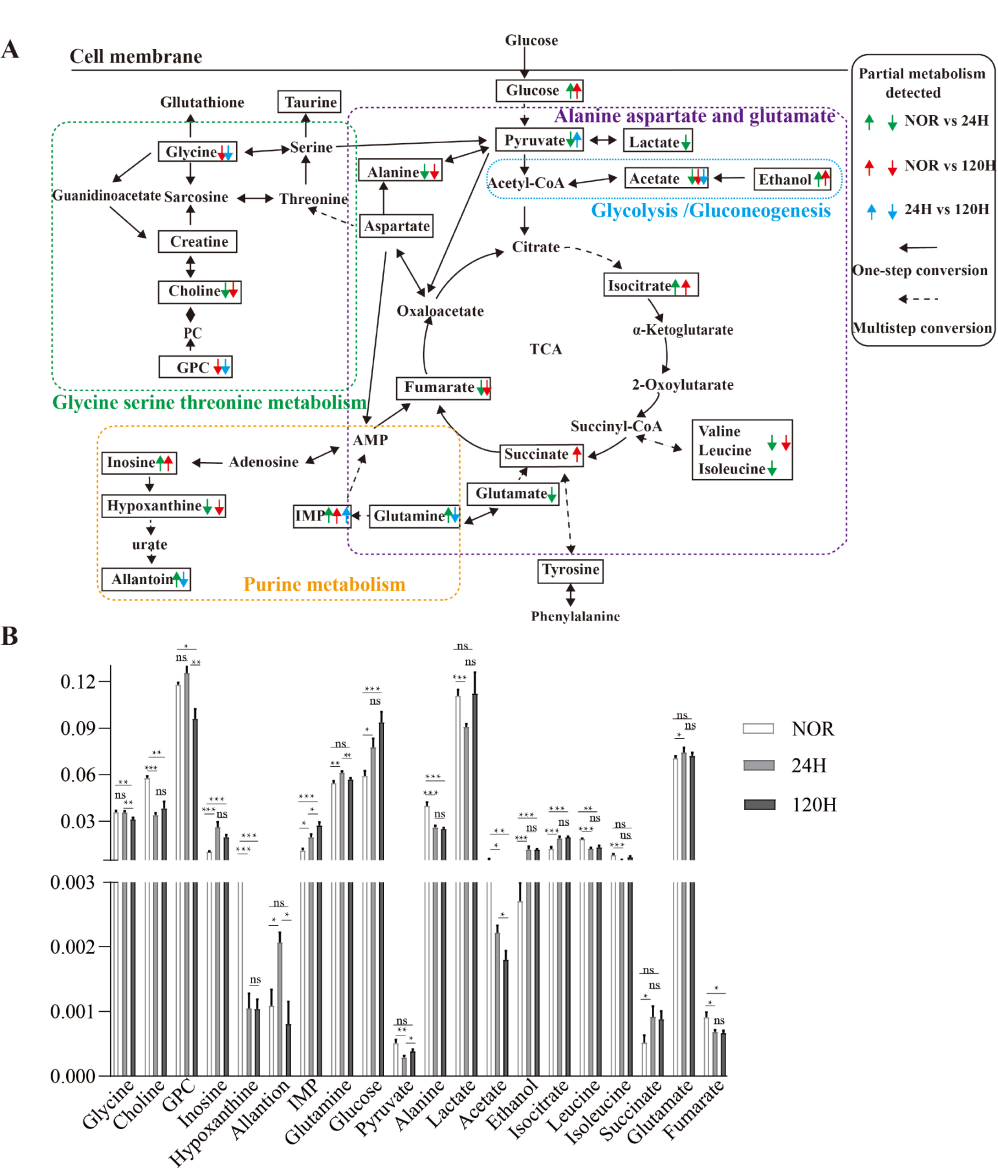
Figure 8 Schematic representation of major metabolic pathway shifts in three sets of mouse lung tissues The metabolic pathways were identified via pairwise comparisons between the NOR and the 24-h, NOR and 120-h, and 24-h and 120-h groups. The up/downwards arrows indicate significantly increased/decreased metabolite levels compared with those in the control group. The dashed arrows represent multiple biochemical reactions, whereas the solid arrows represent individual biochemical reactions. Significantly altered metabolic pathways were determined via the Kyoto Encyclopedia of Genes and Genomes (KEGG) database and the MetaboAnalyst webserver. (B) The significant increases and decreases in metabolite levels are shown in the bar charts. n = 10. ns: not significant. *P < 0.05, **P < 0.01, ***P < 0.001.

On the basis of the above data, we selected three representative comparisons for further analysis, namely, NOR vs 24-h, NOR vs 120-h, and 24-h vs 120-h. These comparisons revealed significant changes in the glycolysis/gluconeogenesis pathway; the purine metabolism pathway; the alanine, aspartate, and glutamate metabolism pathways; and the glycine, serine, and threonine metabolism pathways, as shown in the metabolic pathway diagram ( Figure 8A). The characteristic metabolites in significant metabolic pathways were further analyzed in lung tissue ( Figure 8B). These results suggest that tumor cells can protect them from oxidative stress and provide energy metabolism by altering the metabolic profile of the lung tissue environment, particularly by regulating oxidative metabolism and amino acid metabolism.

Pearson correlation analysis was conducted to examine the relationship between platelet activation and metabolic profile changes in lung metastasis models across the NOR, 24-h, and 120-h groups. The results revealed that platelet-activating factors were positively correlated with several metabolites, including glycine, glycerylphosphorylcholine (GPC), inosine, lactate, and glutamate, and negatively correlated with allantoin, pyruvic acid, acetate, isoleucine, and fumarate ( Supplementary Figure S4). In contrast, the platelet inhibitory factor was positively correlated with allantoin, ethanol, isoleucine, and fumarate and negatively correlated with glycine, glucose, glutamine, lactate, and GPC. These findings provide valuable insights into the associations between platelet activation, protein expression, and metabolic changes during tumor lung metastasis.

### Identification of potential biomarkers associated with tumor metastasis

**Figure 9:**
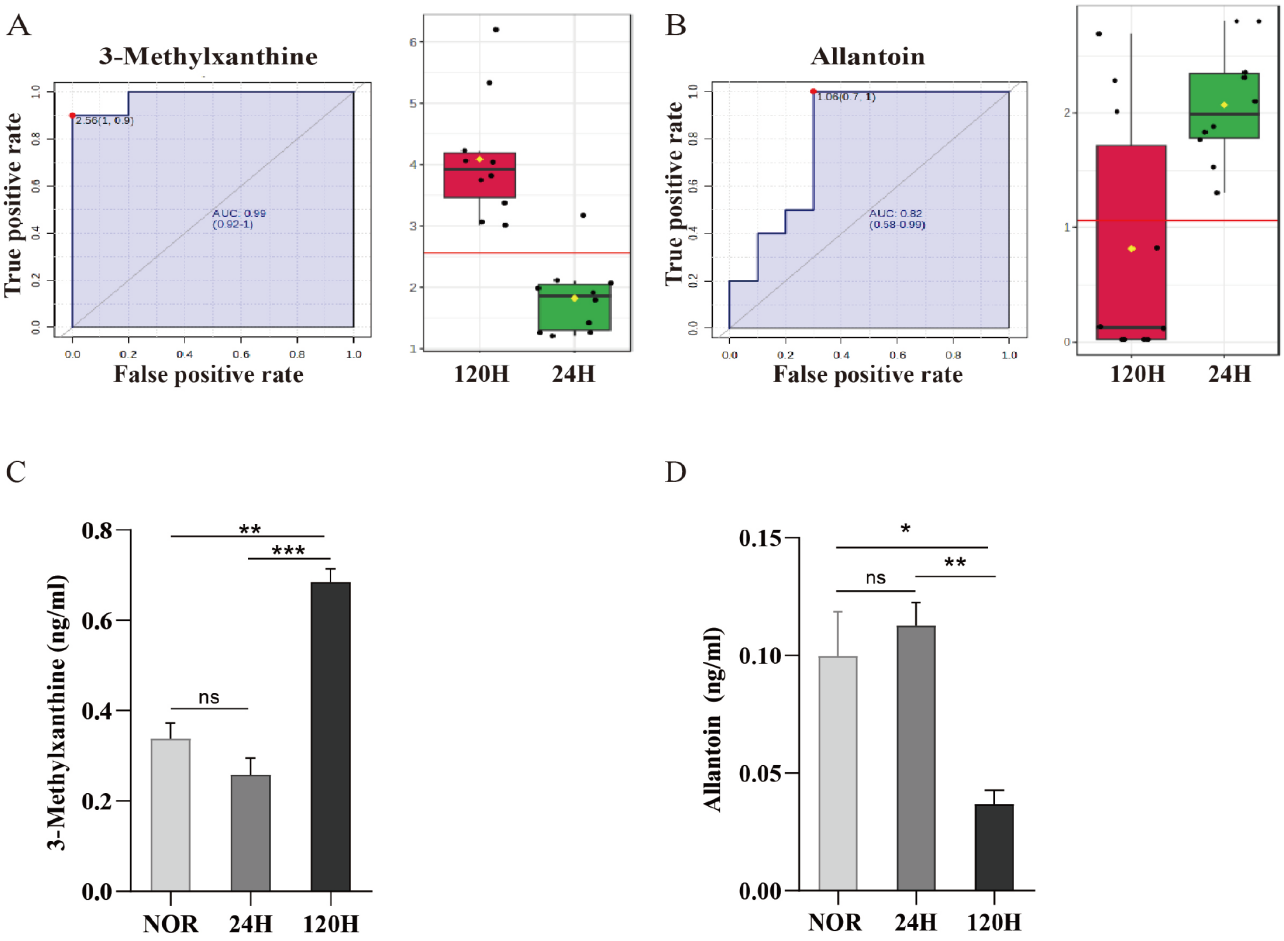
Figure 9 Multivariate ROC analysis and ELISA were performed on the basis of differentially abundant metabolite levels (A,B) AUC values of 0.98 (CI: 0.92–1.0) and 0.82 (CI: 0.58–0.99). (C,D) ELISA analysis of 3-methylxanthine and allantoin expression levels in lung tissue. n = 4. ns, not significant. *P < 0.05, **P < 0.01, ***P < 0.001.

To identify potential biomarkers for distinguishing between the 24-h and 120-h groups, which can represent important time points of tumor metastasis, we conducted ROC analysis on the basis of the relative concentrations of two sets of differentially abundant metabolites ( Supplementary Table S5). Methylxanthine and allantoin were recognized as special biomarkers with AUC values of 0.98 (CI: 0.92–1.0) and 0.82 (CI: 0.58–0.99), respectively ( Figure 9A,B). The ELISA results indicated that the levels of 3-methylxanthine and allantoin remained relatively unchanged at 24 h postinoculation. However, between 24 and 120 h, 3-methylxanthine levels significantly increased, whereas allantoin levels significantly decreased ( Figure 9C,D). These findings suggest that methylxanthine and allantoin may serve as potential biomarkers for differentiating between pre- and postmetastasis stages of tumor cells with high sensitivity and specificity in identifying successful tumor cell engraftment.

## Discussion

Tumor metastasis plays a pivotal role in cancer progression, often leading to recurrence and mortality, particularly in cases of lung metastasis. A comprehensive understanding of the metabolic alterations and molecular mechanisms involved in early-stage lung metastasis is essential for accurate diagnosis and effective treatment of this disease. Although the tumor-node-metastasis (TNM) staging system provides valuable information on the clinical progression of cancer, it lacks the capacity to distinguish between subclinical stages of early metastasis in patients. Furthermore, relying only on clinical imaging is insufficient for detecting pathological changes prior to the formation of metastatic tumors. Therefore, it is necessary to develop sensitive, simple, and reliable biomarkers to evaluate early tumor progression, offering more accurate and personalized treatment strategies for cancer patients.

In this study, we developed an early lung metastasis mouse model of cutaneous melanoma (SKCM-M) to investigate metabolic characteristics during the early stages of metastatic progression and identify potential biomarkers for early metastatic development. The mice were divided into five groups: the NOR, 6-h, 24-h, 72-h, and 120-h groups. Histological H&E staining revealed significant melanoma cell colonization around lung blood vessels in the 72-h and 120-h groups. Moreover, the expressions of regulatory factors of angiogenesis, namely, VEGF and αVβ3, were increased, indicating significant metabolic differences compared with that in the NOR group. Metabolic differences were also detected between the 6-h and 24-h groups, suggesting that tumor cells can adapt to diverse microenvironments throughout the metastatic process, which likely involves reprogramming metabolic pathways in both the primary tumor and distant organs to facilitate metastasis and the development of secondary tumor growth. Moreover, early-stage upregulation of platelet activation factors (CLEC-2, PI3K, and AKT) during tumor metastasis, coupled with decreased expressions of platelet activation inhibitory factors (SHIP-1 and PECAM-1), was identified. These findings suggest that circulating tumor cells trigger platelet activation, creating a protective shield that helps tumor cells evade immune surveillance. These activated platelets also appear to assist in tumor cell adhesion to the vascular endothelium, enabling the tumor cells to use nutrients from the new transplant environment and enhancing metastatic colonization [29].

Metabolic pathway analysis revealed significant disruptions in energy metabolism, particularly in the TCA cycle, glycolysis/gluconeogenesis, and ketone metabolism, as well as disturbances in amino acid metabolism, including the alanine, aspartate, glutamate, glutamine, glycine, serine, and threonine pathways. These metabolic disorders play crucial roles in the early stages of tumor metastasis (Figures 7, 8 , and Supplementary Table S4). However, metabolic characteristics vary across different phases of metastasis, and cancer patients may exhibit varying levels of energy metabolism efficiency [30].

Pyruvate is a metabolite that can be converted to lactate by lactate dehydrogenase. Previous studies have demonstrated that the resistance of circulating tumor cells to oxidative stress is increased by increasing their uptake of lactate and pyruvate, thereby improving their survival [31]. Once tumor cells reach distant organs, they must adapt to the new environment and establish a suitable niche for proliferation. For example, compared with plasma, lung interstitial fluid has a higher concentration of pyruvate. Additionally, the lung is an organ with a greater propensity for tumor cell metastasis in numerous types of cancer [30]. In our experiments, we observed that as tumor cells progressed through metastasis, the levels of pyruvate and lactate gradually decreased, indicating that tumor cells increased their utilization of these metabolites. The biosynthesis and catabolism of alanine rely on reversible reactions catalyzed by ALT1 (cytoplasmic) or ALT2 (mitochondrial), converting alanine and α-ketoglutarate into pyruvate and glutamate. Our findings suggest that energy is continuously consumed during tumor metastasis, leading to a disruption in energy metabolism due to an imbalance between energy intake and utilization.

During metastatic colonization, tumor cells can increase ATP availability by utilizing various nutrients. Notably, ATP produced through glucose metabolism is critical for providing energy during the progression of tumor cell colonization in mouse lungs. Unlike normal cells, tumor cells preferentially use environmental nutrients and rely on aerobic glycolysis for glucose metabolism, even in oxygen-rich conditions [32]. Branched-chain amino acids (BCAAs) are essential nutrients for tumor growth, are involved in multiple biosynthetic pathways, and serve as energy sources [33]. In the early stage of tumor metastasis, free amino acids such as glutamine produce α-ketoglutarate via deamination, which enters the TCA cycle to generate NADH/FADH2, thereby supporting mitochondrial oxidative phosphorylation and cellular energy production. The reduced consumption of BCAAs suggests that their carbon skeletons are extensively utilized for energy metabolism. The hypoxic microenvironment forces cells to rely on glycolysis during the metastasis and colonization stages. Moreover, BCAAs are catalyzed by BCAT1/2 to produce acetyl-CoA, sustaining the production of TCA intermediates and ATP synthesis. The intermediate product of glycolysis, 3-phosphoglycerate (3-PG), combines with the serine synthesis pathway, supplying substrates for one-carbon metabolism and promoting nucleotide biosynthesis. Additionally, TCA intermediates such as citrate and succinate contribute to the regulation of epithelial–mesenchymal transition (EMT)-related gene expression by regulating histone acetylation and DNA methylation, thereby promoting the expansion of metastatic foci. This was demonstrated in our study by the reduced levels of these amino acids (leucine, valine, isoleucine) ( Supplementary Table S3), highlighting the critical role of amino acid metabolism in cancer metastasis progression. Therefore, identifying biomarkers associated with amino acid metabolism may help detect the early stages of metastasis in cancer patients.

We employed ROC analysis, along with univariate and multivariate methods, to identify potential biomarkers for distinguishing the stage of tumor cell colonization at the pre- and postcolonization stages. The selection criteria focused on metabolites that showed significant concentration changes between 24 h and 120 h and had higher VIP scores and higher AUC values. Methylxanthine and allantoin have emerged as potential biomarkers for distinguishing between 24-h and 120-h time points. Specifically, methylxanthine levels in lung tissue increased significantly from 24 h to 120 h, indicating changes in nucleotide metabolism. Methylxanthine can function as an antagonist of adenosine receptors, leading to the inhibition of T-cell activity and the formation of an immunosuppressive microenvironment to promote metastasis and colonization. Conversely, allantoin, known for its therapeutic and preventive effects on cancer, decreased during this period, reflecting enhanced tumor cell metastasis ( Supplementary Table S5). Allantoin protects metastatic cells from oxidative stress damage by clearing ROS while inhibiting dendritic cell maturation and weakening the antitumor immune response. Methylxanthine had a high AUC value of 0.98, indicating its excellent ability to distinguish between the 24-h and 120-h stages ( Figure 9). These findings suggest that methylxanthine and allantoin have potential as biomarkers for monitoring the early progression of tumor metastasis in cancer patients. However, further analysis revealed that neither methylxanthine nor allantoin could serve as biomarkers to distinguish between the 120-h and NOR groups ( Figure 9).

Acetate, similar to glucose, serves as an energy substrate in mammals and is converted into acetyl-CoA for energy production and lipid metabolism. Acetate has gained attention as an alternative energy source for many kinds of tumor cells and is the primary contributor to acetyl-CoA production under stress conditions [34]. In this study, we found that acetate levels in lung tissue decreased within the transition period of early metastasis. During the early stages of metastasis, tumor cell growth is in its initial stage, and tumor cells use acetate to provide energy, while glucose is trapped at high levels due to chronic inflammation and insulin resistance [ 35, 36] . The decrease in acetate levels and increase in glucose levels in early-stage metastatic lung tissue may be related to the conversion of energy supply substrates and energy demands at different stages of cancer progression. Acetate may act as an alternative energy source for tumor cells during periods of stress and high energy requirements. Therefore, acetate level could be a potential biomarker for detecting early-stage tumor metastasis, progression, and severity in cancer patients.

Myo-inositol is an important intracellular signaling molecule that plays a crucial role in maintaining cell volume and osmotic pressure homeostasis. It participates in various cellular processes, including growth, differentiation, and apoptosis. The increase in myo-inositol level may serve as a biomarker for tumor cell proliferation. The proliferative activity of tumor cells can be indirectly assessed by detecting changes in inositol levels [37]. In this study, myo-inositol levels were significantly increased in the 72-h and 120-h groups, indicating an active proliferative state of tumor cells during these periods. Moreover, the expression levels of myo-inositol, inosinic acid, and inosine increased, whereas hypoxanthine level decreased. This result suggested that tumor cells enhanced the pentose phosphate pathway during metastatic progression, supplying additional energy to support their growth and spread. These insights are crucial for understanding tumor metabolism and may promote the development of new therapeutic strategies.

Choline and phosphatidylcholine are important components of the cell membrane, especially choline, which plays a crucial role in its formation and structural integrity. Previous studies have shown that abnormal choline metabolism is a significant metabolic hallmark strongly linked to tumor development. In many malignant cases, choline is extensively converted into phosphocholine [38]. The decrease in phosphocholine level observed in this study may have resulted from enhanced lipid metabolism in metastatic melanoma or the acidic tumor microenvironment, which could restrict its synthesis. Significant changes in methylxanthine and allantoin were observed between the 24- and 120-h time points, suggesting that these compounds may play critical roles in early-stage melanoma metastasis. These findings indicate that methylxanthine and allantoin have the potential to serve as tissue biomarkers for distinguishing early stages of tumor metastasis. Through comprehensive analysis of tumor cell metabolism, we revealed a strong association between metabolic reprogramming and tumor biological behavior, providing new insights and potential strategies for clinical diagnosis and treatment.

The correlations between platelet activation and metabolic profile changes provide valuable insights into the intricate relationship between host metabolism and metastatic progression in the lung. In our study, platelet-activating factors were positively correlated with metabolites such as glycine, GPC, inosine, lactate, and glutamate, which are involved in energy metabolism, nucleotide turnover, and membrane remodeling. These associations suggest that platelet activation may promote a favorable metabolic environment for tumor cell survival, colonization, and proliferation. In contrast, negative correlations with metabolites such as allantoin, pyruvic acid, acetate, isoleucine, and fumarate may reflect platelet-mediated suppression of specific catabolic or redox-related pathways. Notably, the inhibition of platelet activity appeared to counteract the metabolic reprogramming typically associated with tumor metastasis. These findings highlight a functional link between platelet signaling and metabolic adaptation during lung metastasis, indicating that platelets contribute not only to the physical support of circulating tumor cells but also to shaping the tumor microenvironment through metabolic modulation. Targeting the metabolic pathways influenced by platelet signaling may offer a novel therapeutic strategy to disrupt metastatic progression and enhance the clinical utility of metabolite signatures in patient-derived samples.

While our study identified methylxanthine and allantoin as potential biomarkers associated with early-stage melanoma lung metastasis in a murine model, we acknowledge that these findings are currently limited to the preclinical stage. The translational value of these metabolites remains to be confirmed in human patients. Notably, both methylxanthine and allantoin are small, stable molecules, and their detectability supports their potential as candidates for non-invasive diagnostic applications. Therefore, further validation using clinical samples, such as plasma, urine, or exhaled breath condensate from melanoma patients at various metastatic stages, is essential. Future studies should aim to assess the detectability, specificity, and prognostic utility of these metabolites in human biofluids. Validating these metabolites in clinical settings will be critical for determining their feasibility as non-invasive biomarkers for the early detection or monitoring of metastatic progression. Future studies identifying metabolites as biomarkers of early metastasis in humans could lead to the development of screening assays for high-risk patients. Additionally, monitoring these metabolites longitudinally may help clinicians make real-time assessments of metastatic risk. Furthermore, the associations of these metabolites with platelet activation and energy metabolism highlight potential links to tumor cell survival and colonization mechanisms, which may further inform therapeutic strategies. These findings provide a foundation for translational research aimed at developing metabolomics-based, noninvasive diagnostic tools that could be integrated into routine oncology workflows to improve early diagnosis and surveillance of metastatic disease.

## Supporting information

25239Supplementary

## Supplementary Data

Supplementary data are available at *Acta Biochimica et Biophysica Sinica* online.
