# Supplementary material for: Metabolic analysis and identification of potential biomarkers of early-stage melanoma lung metastasis: Metabolic profiling of early-stage melanoma lung metastasis
Source: Acta Biochim Biophys Sin (Shanghai). 2025 Oct 9;58(5):1102–18. doi: 10.3724/abbs.2025136 (PMC13191476; doi:10.3724/abbs.2025136)
Supplement: 25239Supplementary [file 25239Supplementary.docx]

**Supplementary Table S1. Sequences of primers used in quantitative RT-PCR**

| Primer name | Sequence (5′→3′) |
| --- | --- |
| Mouse-CLEC-2/CLEC1b | F: GCTCTGGGGATCATGTCGGT |
|  | R: TATCTCCACTTCGTGGCGCA |
| Mouse-SHIP-1/inpp5d | F: AGTCTTACCCGCTGGTGCAT |
|  | R: ACAGTGCCAGGACCATTCTTG |
| Mouse-PECAME-1/CD31 | F: GGCCAAACAGAAACCCGTGG |
|  | R: AGCCTTCCGTTCTTAGGGTCG |
| Mouse-AKT | F: GCCGCCTGATCAAGTTCTCC |
|  | R: GCTGCTGCCCACAGTAGAAAC |
| Mouse-αVβ3/Itgav | F: GTGCCAGCCCATTGAGTTTG |
|  | R: TGGAGCACAGGCCAAGATTT |
| Mouse-PI3K | F: TATTTGGACTTTGCGACAAGACT |
|  | R: TCGAACGTACTGGTCTGGATAG |
| Mouse-MUS-actin | F: ATGCCACAGGATTCCATACCCAAG |
|  | R: CTCTAGACTTCGAGCAGGAGATGG |

**Supplementary Table S2. Assignment of metabolites extracted from mouse lung tissues using ^1^H-NMR spectra**

| No. | Metabolite | δ^1^H (ppm) and multiplicity |
| --- | --- | --- |
| **1** | Isoleucine (Ile) | 0.94(t),1.01(d),1.21(m),1.42(m),2.00(m),3.67(d) |
| **2** | Leucine (Leu) | 0.96(d),0.97(d),1.69(m),1.70(m),1.73(m),3.73(m) |
| **3** | Valine (Val) | 0.99(d),1.05(d),2.26(m),3.60(d) |
| **4** | Ethanol (Eth) | 1.17(t),3.65(q) |
| **5** | Lactate (Lac) | 1.33(d),4.11(q) |
| **6** | Alanine (Ala) | 1.47(d),3.78(q) |
| **7** | Acetate (Ac) | 1.90 (s) |
| **8** | Glutamate (Glu) | 2.08(m),2.12(m),2.34(m),2.37(m),3.75(m) |
| **9** | Glutamine (Gln) | 2.13(m),2.45(m),3.77(t) |
| **10** | Aspartate (Asp) | 2.68(dd), 2.81(dd), 3.90(dd) |
| **11** | Isocitrate (ISO) | 2.55(m), 2.98(dd), 4.05(d) |
| **12** | Creatine (Cr) | 3.04(s),3.93(s) |
| **13** | Malonate | 3.11(s) |
| **14** | Taurine (Tau) | 3.26(t),3.43(t) |
| **15** | Methanol | 3.37（s） |
| **16** | Glycine (Gly) | 3.57(s) |
| **17** | Sn-giycero-3-phosphocholine(Gpc) | 3.23 (s), 3.60 (dd), 3.68 (dd), 3.87 (m), 3.94 (m), 4.33 (m) |
| **18** | Myo-inositol (Ins) | 3.3(t), 3.55 (dd), 3.63(t), 4.07(t) |
| **19** | Glucose (Glc) | 3.29 (dd), 3.48(t), 3.54 (dd), 3.71 (t), 3.72 (dd),3.83 (m), 3.901(dd), 4.65(d),  5.30 (d) |
| **20** | IMP | 4.0(m),4.37(m),4.52(q),6.15(d),8.23(s),8.59(s) |
| **21** | Inosine (Ino) | 3.83(d),3.84(d),4.28(dd),4.44(t),6.1(d),8.23(s),8.35(s) |
| **22** | Choline | 3.2(s), 3.52 (dd), 4.03(t) |
| **23** | Urea | 5.81(s) |
| **24** | Fumarate (Fum) | 6.52(s) |
| **25** | ATP | 6.14(d),8.27(s),8.58(s) |
| **26** | ADP | 6.13(d),8.27(s),8.58(s) |
| **27** | Niacinamide (NA) | 7.60(dd),8.25(dd),8.72(dd),8.92(s) |
| **28** | 3-Methylxanthine (3Mx) | 3.53(s),8.0(s) |
| **29** | Oxypurinol | 8.19(s) |
| **30** | Formate | 8.46(s) |
| **31** | Uracil (Ura) | 5.81(d),7.54(d) |
| **32** | Allantoin | 5.38(s) |
| **33** | Ump | 3.40(m),4.28(m),4.38(m),4.44(m),5.99(m),8.1(m) |
| **34** | Succinate | 2.41(s) |
| **35** | Pyruvate (Pyr) | 2.38(s) |
| **36** | dTTP | 1.92(s),4.17(t),7.68(s) |
| **37** | 4-Hydroxybenzoate | 6.9(d),7.81(dd) |
| **38** | Tyrosine (Tyr) | 3.05(dd),3.19(dd),3.96(dd),6.92(d),7.19(d) |
| **39** | Hypoxanthine (Hyp) | 8.2(s) |
| **40** | Phenylalanine (Phe) | 3.12(dd),3.30(dd),3.99(dd),7.33(d),7.37(t),7.43(t) |

s, singlet; d, double; t, triplet; q, quartet; m, multiple; dd, double of double. Abbreviations include: ATP, Adenosine Triphosphate; ADP, Adenosine Diphosphate; IMP, Inosine Monophosphate; UMP, Uridine Monophosphate; DTTP, Deoxythymidine Triphosphate.

**Supplementary Table S3. Information of assigned metabolites in 1D ^1^H-NMR spectra**

|  | Mean ± SD | | | | | NOR | NOR | NOR | NOR | ANOVA | |
| --- | --- | --- | --- | --- | --- | --- | --- | --- | --- | --- | --- |
| Meta bolite | NOR | 6H | 24H | 72H | 120H | vs. 6H | vs. 24H | vs. 72H | vs. 120H | F | *P* |
| Isoleucine | 0.00836±0.00199 | 0.00690±0.00195 | 0.00514±0.00077 | 0.00416±0.00078 | 0.00682±0.00242 | ns | ** | *** | ns | 9.072 | <0.0001 |
| Leucine | 0.01861±0.00099 | 0.01143±0.00394 | 0.01244±0.00180 | 0.01348±0.00214 | 0.01331±0.00331 | *** | *** | *** | ** | 10.94 | <0.0001 |
| Valine | 0.00896±0.00259 | 0.00754±0.00089 | 0.00747±0.00196 | 0.00626±0.00064 | 0.00820±0.00076 | ns | ns | ** | ns | 4.055 | 0.0068 |
| Ethanol | 0.00271±0.00089 | 0.01261±0.00144 | 0.01186±0.00676 | 0.00899±0.00359 | 0.01188±0.00094 | *** | ** | *** | *** | 13.48 | <0.0001 |
| Lactate | 0.11083±0.01242 | 0.09296±0.02309 | 0.09073±0.00664 | 0.06459±0.00856 | 0.11227±0.04301 | * | ** | *** | ns | 7.14 | 0.0001 |
| Alanine | 0.04000±0.00730 | 0.03245±0.00688 | 0.02613±0.00350 | 0.02502±0.00309 | 0.02508±0.00257 | * | *** | *** | *** | 16.43 | <0.0001 |
| Acetate | 0.00487±0.00325 | 0.00329±0.00209 | 0.00222±0.00038 | 0.00146±0.00015 | 0.00180±0.00044 | ns | * | ** | ** | 6.248 | 0.0004 |
| Glutamate | 0.07061±0.00438 | 0.06621±0.00441 | 0.07432±0.01004 | 0.06931±0.00363 | 0.00903±0.00381 | ns | * | ns | ns | 2.393 | 0.0646 |
| Glutamine | 0.05459±0.00440 | 0.05021±0.00289 | 0.06126±0.00332 | 0.05680±0.00212 | 0.05691±0.00304 | * | ** | ns | ns | 15.43 | <0.0001 |
| Aspartate | 0.02591±0.00172 | 0.02479±0.00409 | 0.02698±0.00780 | 0.02788±0.00309 | 0.02545±0.00485 | ns | ns | ns | ns | 0.6672 | 0.6181 |
| Isocitrate | 0.01234±0.00378 | 0.01791±0.00778 | 0.01903±0.00381 | 0.02141±0.00193 | 0.01970±0.00181 | ns | ** | *** | *** | 6.182 | 0.0005 |
| Creatine | 0.01968±0.00693 | 0.01303±0.00065 | 0.01509±0.00156 | 0.01286±0.00068 | 0.01743±0.00448 | ** | ns | ** | ns | 6.014 | 0.0006 |
| Malonate | 0.00399±0.00120 | 0.00397±0.00097 | 0.00308±0.00068 | 0.00331±0.00164 | 0.00411±0.00139 | ns | ns | ns | ns | 1.438 | 0.237 |
| Taurine | 0.18890±0.01483 | 0.16522±0.00839 | 0.18951±0.01677 | 0.19300±0.00724 | 0.19257±0.00842 | ** | ns | ns | ns | 9.796 | <0.0001 |
| Glycine | 0.03597±0.00287 | 0.03290±0.00340 | 0.03573±0.00232 | 0.03274±0.00225 | 0.03128±0.00349 | * | ns | * | ** | 4.917 | 0.0022 |
| Sn-giycero-3-  phosphocholine | 0.11788±0.00441 | 0.09268±0.02294 | 0.12549±0.02741 | 0.10293±0.00396 | 0.09602±0.01960 | ** | ns | *** | ** | 5.914 | 0.0007 |
| Myo-inositol | 0.03699±0.00637 | 0.03330±0.00703 | 0.03954±0.00445 | 0.04253±0.00237 | 0.04411±0.00814 | ns | ns | * | * | 5.163 | 0.0016 |
| Glucose | 0.05920±0.01018 | 0.14542±0.03071 | 0.07768±0.01768 | 0.11083±0.00758 | 0.09380±0.02067 | *** | * | *** | ** | 29.47 | <0.0001 |
| IMP | 0.01119±0.00410 | 0.01192±0.00612 | 0.01984±0.00580 | 0.02874±0.00356 | 0.02738±0.00630 | ns | ** | *** | *** | 24.37 | <0.0001 |
| Inosine | 0.01051±0.00079 | 0.02244±0.00585 | 0.02620±0.01079 | 0.02212±0.00270 | 0.01970±0.00506 | *** | ** | *** | *** | 9.428 | <0.0001 |
| Choline | 0.05792±0.00393 | 0.05581±0.00856 | 0.03400±0.00468 | 0.03284±0.00775 | 0.03836±0.01385 | ns | *** | *** | ** | 20.32 | <0.0001 |
| Urea | 0.00219±0.00554 | 0.00187±0.00484 | 0.01081±0.01480 | 0.01632±0.00315 | 0.00539±0.00477 | ns | ns | *** | ns | 6.248 | 0.0004 |
| Fumarate | 0.00091±0.00024 | 0.00094±0.00031 | 0.00068±0.00011 | 0.00065±0.00009 | 0.00067±0.00012 | ns | * | ** | * | 5.211 | 0.0016 |
| ATP | 0.01695±0.00433 | 0.01263±0.00515 | 0.01474±0.00356 | 0.01549±0.00282 | 0.01096±0.00164 | ns | ns | ns | ** | 4.093 | 0.0065 |
| ADP | 0.01635±0.00318 | 0.01179±0.00412 | 0.01448±0.00308 | 0.01174±0.00253 | 0.00922±0.00191 | * | ns | ** | *** | 8.149 | <0.0001 |
| Niacinamide | 0.00424±0.00148 | 0.00352±0.00064 | 0.00259±0.00062 | 0.00309±0.00029 | 0.00470±0.00307 | ns | ** | * | ns | 2.906 | 0.0319 |
| 3-  Methylxanthine | 0.00311±0.00091 | 0.00589±0.00103 | 0.00172±0.00039 | 0.00374±0.00043 | 0.00408±0.00099 | *** | ** | ns | * | 35.86 | <0.0001 |
| Oxypurinol | 0.00165±0.00061 | 0.00152±0.00062 | 0.00080±0.00029 | 0.00087±0.00024 | 0.00084±0.00011 | ns | *** | ** | *** | 9.241 | <0.0001 |
| Formate | 0.00046±0.00024 | 0.00041±0.00025 | 0.00126±0.00160 | 0.00028±0.00006 | 0.00038±0.00011 | ns | ns | * | ns | 2.982 | 0.0288 |
| Uracil | 0.00101±0.00035 | 0.00077±0.00042 | 0.00037±0.00025 | 0.00145±0.00071 | 0.00069±0.00036 | ns | *** | ns | ns | 8.031 | <0.0001 |
| Allantoin | 0.00109±0.00078 | 0.00158±0.00107 | 0.00206±0.00050 | 0.00162±0.00090 | 0.00081±0.00109 | ns | ** | ns | ns | 3.021 | 0.0273 |
| Ump | 0.02001±0.00358 | 0.02494±0.01033 | 0.02314±0.00501 | 0.02498±0.00197 | 0.02212±0.00341 | ns | ns | ** | ns | 1.358 | 0.2634 |
| Succinate | 0.00052±0.00036 | 0.00057±0.00021 | 0.00092±0.00052 | 0.00076±0.00027 | 0.00088±0.00039 | ns | ns | ns | * | 2.414 | 0.0627 |
| Pyruvate | 0.00051±0.00017 | 0.00044±0.00015 | 0.00028±0.00010 | 0.00038±0.00007 | 0.00039±0.00009 | ns | ** | * | ns | 4.663 | 0.0031 |
| DTTP | 0.01195±0.00325 | 0.01110±0.00210 | 0.00748±0.00240 | 0.00875±0.00181 | 0.00560±0.00097 | ns | ** | * | *** | 13.55 | <0.0001 |
| 4-  Hydroxybenzoate | 0.00082±0.00033 | 0.00024±0.00015 | 0.00029±0.00017 | 0.00966±0.00878 | 0.00075±0.00041 | *** | ** | * | ns | 10.81 | <0.0001 |
| Tyrosine | 0.00552±0.00070 | 0.00378±0.00077 | 0.00529±0.00100 | 0.00385±0.00095 | 0.00522±0.00059 | *** | ns | ** | ns | 10.69 | <0.0001 |

The *P*-values were determined by one-way analysis of variance (ANOVA) to assess statistical significance and then subjected to Tukey’s multiple comparison test: ns (not significant), *P* > 0.05; ↑ or ↓ (upward or downward arrow), *P* < 0.05; ↑↑ or ↓↓, *P* <0.01; ↑↑↑ or ↓↓↓, *P* <0.001; ↑↑↑↑ or ↓↓↓↓, *P* < 0.0001. Metabolites with significant differences were identified with *P* <0.05. Upward and downward arrows indicate positive (A increases compared to B), and negative (A decreases compared to B) differences between A and B, respectively.

**Supplementary Table S4. Metabolic pathways in five sets of mouse lung tissues**

| No. | Metabolic Pathway | NOR vs 6 h | NOR vs 24 h | NOR vs 72 h | NOR vs 120 h | 24H vs 120 h |
| --- | --- | --- | --- | --- | --- | --- |
| 1 | Glycolysis / Gluconeogenesis | √ | √ | √ | √ | √ |
| 2 | Tyrosine metabolism | √ |  | √ |  |  |
| 3 | Purine metabolism | √ | √ | √ | √ | √ |
| 4 | Phenylalanine,tyrosine and tryptophan biosynthesis | √ |  | √ |  |  |
| 5 | Taurine and hypotaurine metabolism | √ |  |  |  |  |
| 6 | Alanine, aspartate and glutamate metabolism | √ | √ | √ | √ |  |
| 7 | Glutathione metabolism | √ |  |  |  |  |
| 8 | Pyruvate metabolism | √ | √ | √ |  |  |
| 9 | Glycine, serine and threonine metabolism | √ | √ | √ | √ |  |
| 10 | Citrate cycle (TCA cycle) |  | √ | √ | √ |  |
| 11 | Glycerolipid metabolism |  | √ | √ | √ |  |
| 12 | Pyrimidine metabolism |  | √ | √ | √ | √ |
| 13 | Arginine biosynthesis |  |  | √ |  |  |
| 14 | Inositol phosphate metabolism |  |  |  | √ |  |

Pathway Impact Value (PIV) > 0.1, *P* < 0.05.

**Supplementary Table S5. Potential biomarkers distinguished between the 24-h and 120-h time points**

| Metabolite | ROC analysis | | | Univariate & multivariate analysis | |
| --- | --- | --- | --- | --- | --- |
|  | AUC | *P* | Log_2_ (fold change) | Significance | VIP |
| Acetate | 0.7700 | 0.0413 | -0.3032 | 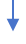 | 1.0758 |
| Glutamine | 0.8600 | 0.0065 | -0.1064 | 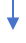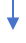 | 1.2430 |
| Valine | 0.7000 | 0.1306 | 0.1348 | ns | 1.0728 |
| Malonate | 0.7300 | 0.0821 | 0.4153 | ns | 1.0087 |
| Glycine | 0.8500 | 0.0082 | -0.1919 | 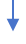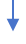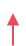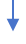 | 1.2696 |
| Gpc | 0.8300 | 0.0126 | -0.3862 |  | 1.2731 |
| IMP | 0.8000 | 0.0233 | 0.4645 |  | 1.1367 |
| Inosine | 0.7200 | 0.0963 | -0.4114 | ns | 0.8987 |
| ATP | 0.8400 | 0.0102 | -0.4277 | 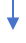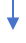 | 1.3051 |
| ADP | 0.9200 | 0.0015 | -0.6516 | 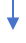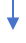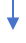 | 1.5510 |
| 3-Methylxanthine | 0.9800 | 0.0003 | 1.1654 | 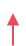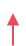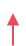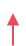 | 1.7063 |
| Formate | 0.7100 | 0.1124 | -1.7512 | ns | 0.7988 |
| Uracil | 0.7500 | 0.0588 | 0.8660 | 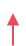 | 1.0560 |
| Allantoin | 0.8200 | 0.0191 | -1.3387 | 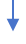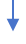 | 1.3098 |
| Pyruvate | 0.7300 | 0.0821 | 0.4422 | 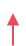 | 1.2164 |
| 4-Hydroxybenzoate | 0.9200 | 0.0019 | 1.3207 | 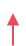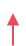 | 1.2142 |
| DTTP | 0.8100 | 0.0191 | -0.4179 | 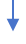 | 0.9950 |

Criteria for the Selection of Potential Biomarkers: High AUC value (AUC > 0.7), significant changes in serum metabolite concentration [Log_2_(fold change) > 0.6 or < −1], and substantial Variable Importance in Projection (VIP > 1) during the transition from 24 hours to 120 hours.


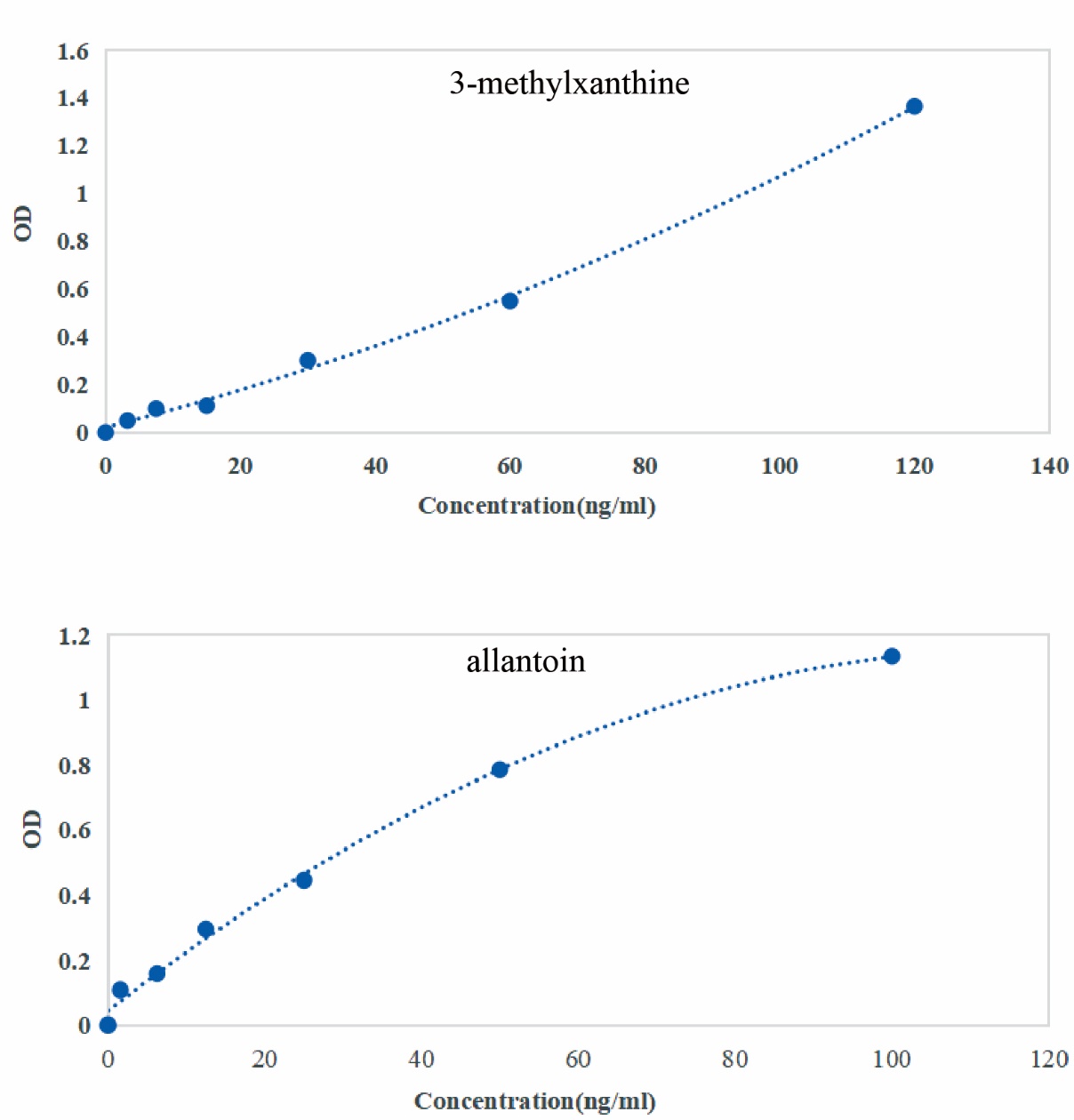


**Supplementary Figure S1.** **ELISA standard curves of 3-methylxanthine and allantoin** The ELISA standard curves were generated with known concentrations according to the manufacturer’s instructions. The concentration values for 3-methylxanthine standard curve: 120, 60, 30, 15, 7.5, 3.75, 0 ng/mL. For allantoin standard curve: 100, 50, 25, 12.5, 6.25, 3.12, 0 ng/mL.


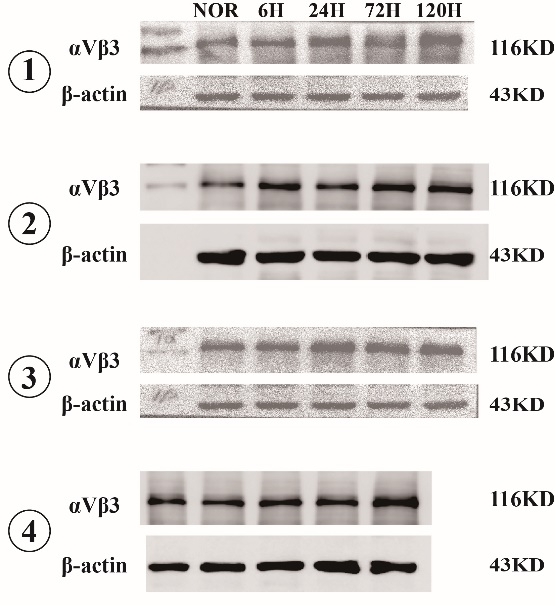

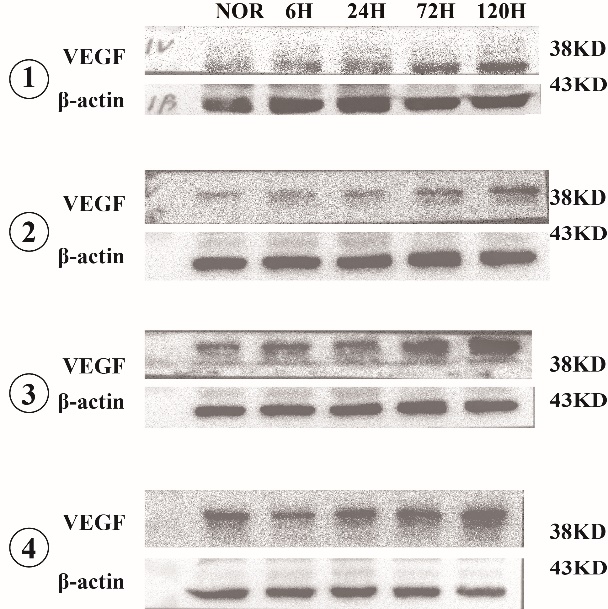


αVβ3 VEGF


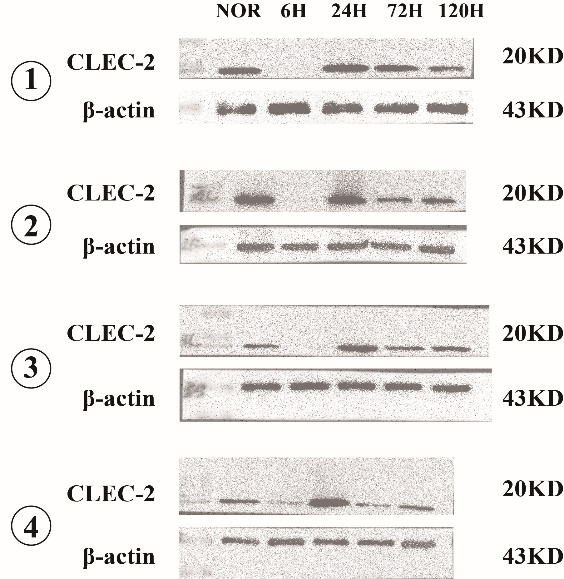

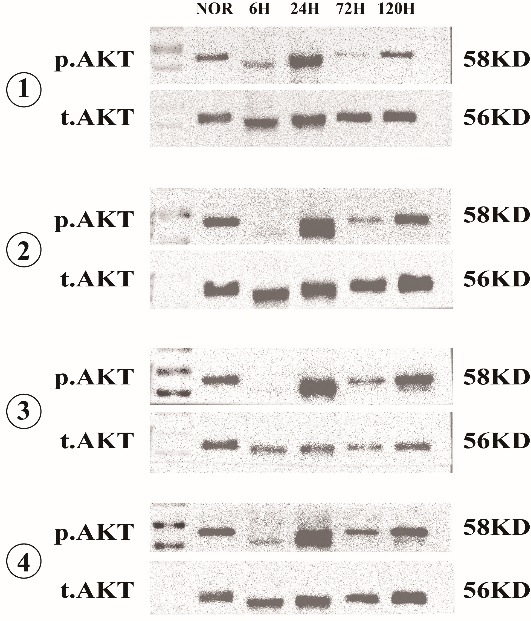


CLEC-2 AKT

**
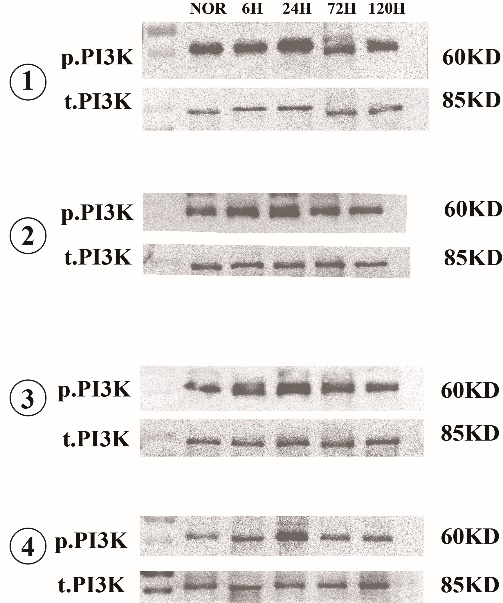

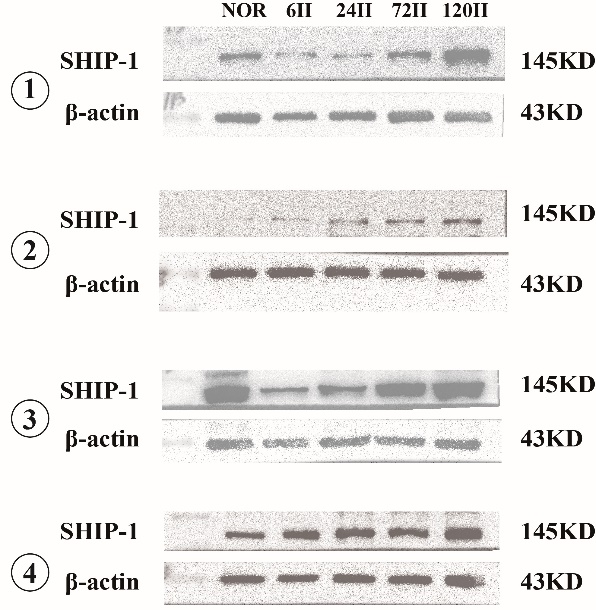
**

PI3K SHIP-1

**
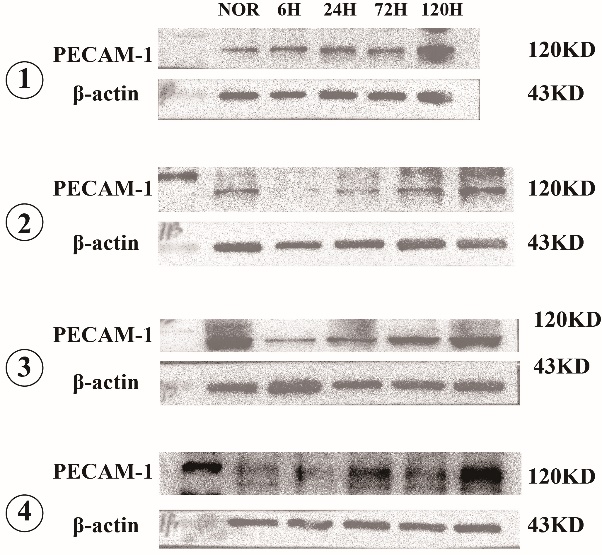
**

PECAM-1

**Supplementary Figure S2.** **Western blots from multiple biological replicates** A representative blot for each kind of factor was selected to show in **Figure 2** and **Figure 3**. Western blots from multiple biological replicates are presented here.


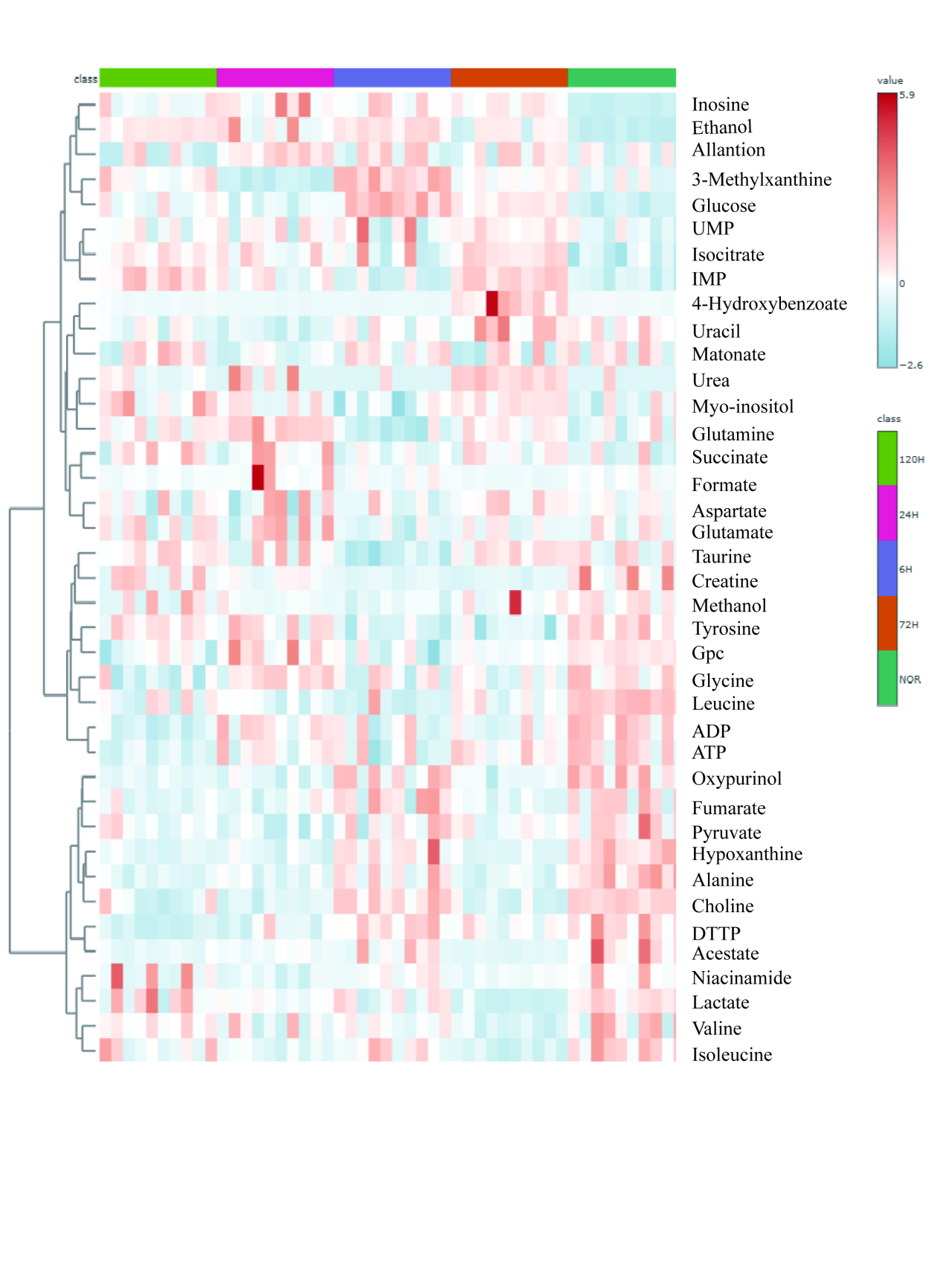


**Supplementary Figure S3. Heatmap of relative levels of metabolites in five sets of mouse lung tissues** There are five groups: NOR, 6-h, 24-h, 72-h, and 120-h groups. This provides a visual representation of changes in metabolite levels.


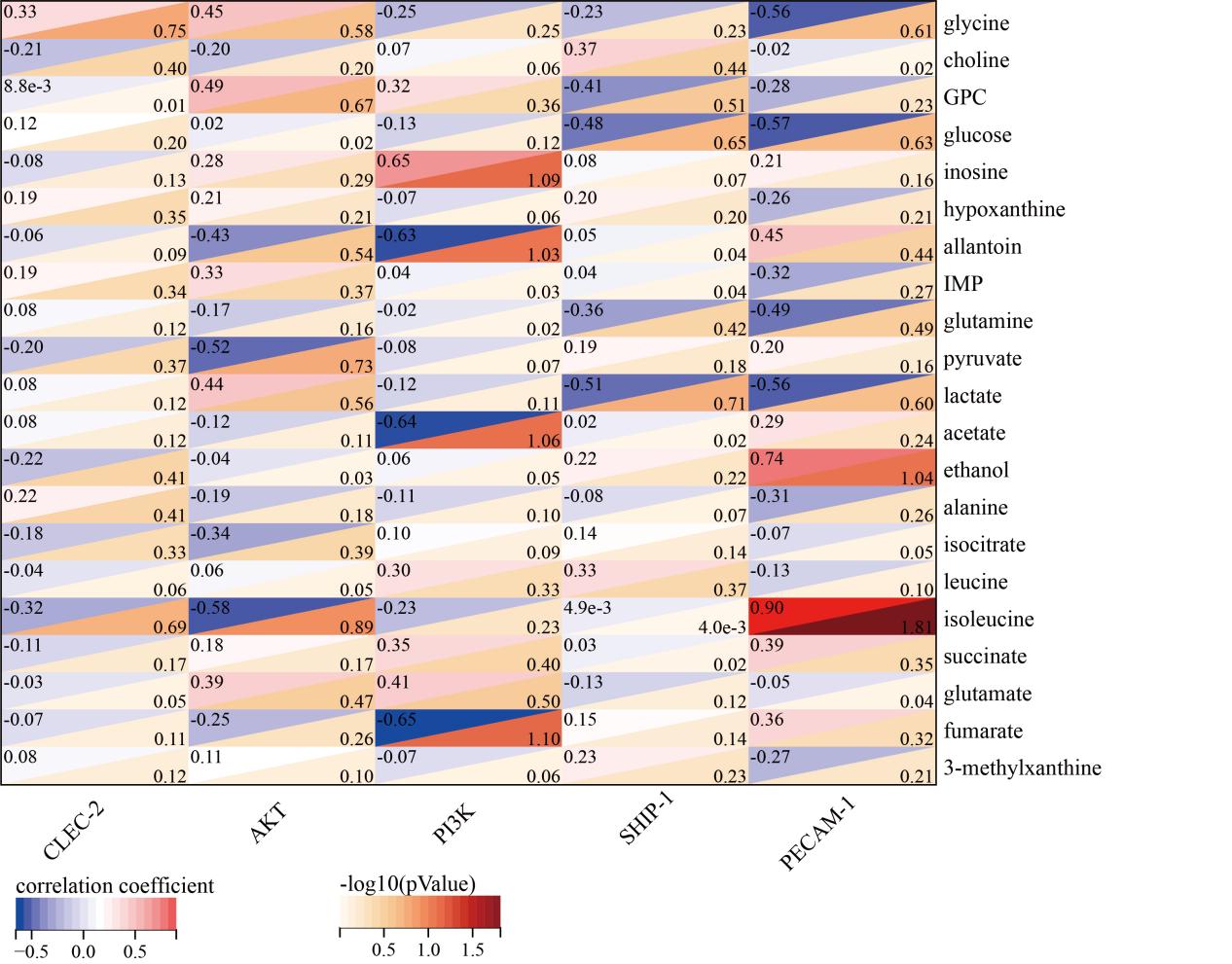


**Supplementary Figure S4. Pearson correlation analysis between platelet-activating factor levels and pulmonary metastatic metabolism** Pearson correlation analysis was performed to assess the relationship between platelet-activating factors and metabolites in lung metastasis models across the NOR, 24-h, and 120-h groups.
